# CRAmed: a conditional randomization test for high-dimensional mediation analysis in sparse microbiome data

**DOI:** 10.1093/bioinformatics/btaf038

**Published:** 2025-01-28

**Authors:** Tiantian Liu, Xiangnan Xu, Tao Wang, Peirong Xu

**Affiliations:** Research Center of Biostatistics and Computational Pharmacy, China Pharmaceutical University, Jiangsu 211198, China; Chair of Statistics, Humboldt-Universität zu Berlin, Berlin 10099, Germany; SJTU-Yale Joint Center of Biostatistics and Data Science, Shanghai Jiao Tong University, Shanghai 200240, China; Department of Statistics, School of Mathematical Sciences, Shanghai Jiao Tong University, Shanghai 200240, China; MOE-LSC & CMA-Shanghai, Shanghai Jiao Tong University, Shanghai 200240, China; MoE Key Lab of Artificial Intelligence, AI Institute, Shanghai Jiao Tong University, Shanghai 200240, China; Department of Statistics, School of Mathematical Sciences, Shanghai Jiao Tong University, Shanghai 200240, China

## Abstract

**Motivation:**

Numerous microbiome studies have revealed significant associations between the microbiome and human health and disease. These findings have motivated researchers to explore the causal role of the microbiome in human complex traits and diseases. However, the complexities of microbiome data pose challenges for statistical analysis and interpretation of causal effects.

**Results:**

We introduced a novel statistical framework, CRAmed, for inferring the mediating role of the microbiome between treatment and outcome. CRAmed improved the interpretability of the mediation analysis by decomposing the natural indirect effect into two parts, corresponding to the presence–absence and abundance of a microbe, respectively. Comprehensive simulations demonstrated the superior performance of CRAmed in Recall, precision, and F1 score, with a notable level of robustness, compared to existing mediation analysis methods. Furthermore, two real data applications illustrated the effectiveness and interpretability of CRAmed. Our research revealed that CRAmed holds promise for uncovering the mediating role of the microbiome and understanding of the factors influencing host health.

**Availability and implementation:**

The R package CRAmed implementing the proposed methods is available online at https://github.com/liudoubletian/CRAmed.

## 1 Introduction

The microbiome plays a pivotal role in human health, with research revealing associations between microbiome dysbiosis and various diseases, including inflammatory bowel disease (Glassner *et al.* 2020), type 2 diabetes (Sharma and Tripathi 2019), and Parkinson’s disease (Jackson *et al.* 2019, Lubomski *et al.* 2022). As research progresses, we have moved beyond establishing mere associations to probing causal relationships. Particularly noteworthy is the recognition of the human microbiome as a crucial causal mediator in the effects of treatments or exposures on health. For instance, Wang *et al.* (2021a) unveiled how modifications in gut microbiota could act as a mediator in the precise dietary intervention to prevent cardiometabolic diseases. Another study shed light on the mediating role of the gut microbiota in the context of total parenteral nutrition and its impact on glucose metabolism disorders (Wang *et al.* 2023). Therefore, a deeper understanding of the microbiome’s mediation role holds the potential of facilitating the translation of microbiome research into precise clinical insights for noninvasive wellness monitoring, diagnosis, and treatment.

Mediation analysis, as a type of causal inference method, offers a statistical framework to explore whether a treatment or exposure influences an outcome by acting through a mediator. Traditional mediation analysis primarily focuses on the univariate model, wherein a single mediator is considered at a time. This approach has found widespread application in fields like social psychology and medicine (Baron and Kenny 1986, MacKinnon *et al.* 2002, VanderWeele 2011). In recent years, there have been advancements in mediation analysis methods that allow for the inclusion of multiple mediators. One notable approach is the MultiMed method proposed by Boca *et al.* (2014). This method uses a permutation approach to simultaneously test multiple mediators while effectively controlling the family-wise error rate. By considering multiple mediators, researchers can gain a more comprehensive understanding of the complex pathways through which treatments or exposures influence outcomes.

The intricacies of microbiome data, including high-dimensionality, sparsity, and compositionality (Knight *et al.* 2018), present challenges to statistical analysis and interpretation. Researchers have developed two primary strategies to deal with high-dimensional mediators: dimension reduction and variable selection. Dimension reduction-based methods aim to condense the information contained in the mediators into a smaller set of components. Techniques such as principal component analysis have been widely used for this purpose (Huang and Pan 2016, Zhao *et al.* 2020). On the other hand, variable selection-based methods focus on identifying the most important variables for mediation analysis through the combination of screening and regularization techniques (Zhang *et al.* 2016, Gao *et al.* 2019, Perera *et al.* 2022). However, simply adopting these methods for microbiome mediation analysis without considering sparsity and compositionality may yield biased and misleading results.

In prior studies, researchers have made efforts to account for the unique characteristics of microbiome data in mediation analysis. For example, recognizing that not all microbial taxa are present in every sample, Wu *et al.* (2022) developed a marginal mediation analysis method to address data sparsity, introducing a zero-inflated Beta distribution to model each mediator separately. However, with hundreds or thousands of taxa, selecting mediators while ensuring finite-sample false discovery rate (FDR) control poses a significant challenge for marginal methods. By using mathematical operations and techniques tailored for compositional data within the simplex space, Sohn and Li (2019) and Sohn *et al.* (2021) proposed sparse compositional mediation models for estimating mediation effects. Wang *et al.* (2020) used a linear log-contrast model and a Dirichlet regression model, incorporating regularization techniques for variable selection. Utilizing isometric log-ratio transformations of relative abundances as mediator variables, Zhang *et al.* (2021a,b) applied de-biased LASSO to estimate mediation effects. One major limitation of these joint mediation methods is their ineffectiveness in handling rare taxa. To address this issue, Yue and Hu (2022) proposed an inverse regression-based approach named LDM-med, which converts the microbial abundance table into a presence–absence matrix. This method offers improved control over the FDR in the presence of rare taxa.

In this article, we propose a novel method named CRAmed for inferring the mediation effects of the microbiome in the relationship between a treatment and an outcome. CRAmed utilizes the zero-inflated negative binomial distribution to describe the microbiome sequencing data, and decomposes the natural indirect effect into two components: the presence–absence status and variation in the abundance. CRAmed then uses a joint significant test to identify the significant mediators. In particular, to address the high dimensionality, we introduce a conditional randomization test. Simulation experiments and analyses of real data are conducted to evaluate the performance of CRAmed and compare it to existing mediation analysis approaches.

## 2 Materials and methods

The input of CRAmed comprises two *n*-vectors, Y= (*Y_i_*) and T= (*T_i_*), along with a *n *×* m* matrix M= (*M_ij_*), i=1,…,n, j=1,…,m, representing *n* observations on a continuous outcome *Y*, a binary treatment or exposure *T*, and *m* microbial mediators M= (*M_j_*), respectively. Our CRAmed also allows for the inclusion of a *n *×* q* matrix X= (*X_ik_*), comprising *q* confounding variables X= (*X_k_*), k=1,…,q, such as baseline covariates like gender and age. Then CRAmed fits two models: one for the regression of *Y* onto M, *T*, and X, and another for the regression of M onto *T* and X. Following this, it uses a zero-inflated conditional randomization test and a Wald test to evaluate the significance of each microbe as a mediator. Finally, for each of the identified mediators, CRAmed calculates the mediation effect, and decomposes the average natural indirect effect by considering the impact of both the presence–absence and abundance of a microbe. We describe details in the following sections.

### 2.1 The mediation model

Mediation models play a crucial role in examining whether an exposure influences an outcome through intermediaries. We adopt the following model for microbiome mediation analysis:

$$\begin{array}{c} M_{j}∼\{\begin{array}{ccc} 0, & & \text{with}\;\text{probability}\;\pi_{j},\\ NB(\lambda_{j},\phi_{j}), & & \text{with}\;\text{probability}\;1-\pi_{j}, \end{array}\\ Y∼N(\mu ,\sigma^{2}), \end{array}$$

where *NB*($\lambda_{j},\phi_{j}$) denotes the negative-binomial distribution with mean *λ_j_* and dispersion $\phi_{j}$, and *N*($\mu ,\sigma^{2}$) represents the normal distribution with mean *μ* and variance $\sigma^{2}$. Moreover, the parameters *π_j_*, *λ_j_*, and *μ* satisfy

$$\begin{array}{c} \text{logit}(\pi_{j})=\gamma_{0j}+\gamma_{1j}T+\gamma_{xj}^{⊤}X,\\ \;\text{log}(\lambda_{j})=\alpha_{0j}+\alpha_{1j}T+\alpha_{xj}^{⊤}X, \end{array}$$

and

$$\mu =\beta_{0}+\beta_{1}T+\beta_{x}^{⊤}X+\beta_{m}^{⊤}M,$$

for coefficients $\gamma_{0j},\;\alpha_{0j}$, *β*_0_, $\gamma_{1j},\;\alpha_{1j}$, *β*_1_, $\gamma_{xj},\;\alpha_{xj},\;\beta_{x}$, and $\beta_{m}$.

### 2.2 Expressions of average natural direct and indirect effects

Let ***M***(*t*) be the counterfactual value of ***M*** if exposure *T* were set to the value *t* and let *Y*(t,m) denote the counterfactual value for *Y* if *T* were set to *t* and ***M*** were set to ***m***. The average natural direct effect (NDE) conditional on X=x is defined as E{Y(1,M(0))–*Y*(0,M(0))| X=x}, and the average natural indirect effect (NIE) conditional on X=x is defined as E{Y(1,M(1))– *Y*(1,M0)| X=x}.

The identification of average NDE and average NIE requires the following assumptions:

**A1**: no unmeasured confounders for the relationship between treatment and outcome, i.e. *Y*(t,m) ⊥⊥ T | X for all levels of *t* and ***m***;

**A2**: no unmeasured confounders for the relationship between mediator and outcome, i.e. *Y*(t,m) ⊥⊥ M | T,X for all levels of *t* and ***m***;

**A3**: no unmeasured confounders for the relationship between treatment and mediator, i.e. ***M***(*t*) ⊥⊥ T | X for all levels of *t*;

**A4**: no unmeasured confounders for the relationship between mediator and outcome that can be affected by the treatment, i.e. *Y*(t,m) ⊥⊥ M(t′) | X for all levels of *t*, t′, and ***m***.

Additionally, it is assumed that the mediators are not causally related, meaning that one mediator cannot be the cause of another. For a discussion of these fundamental assumptions, see VanderWeele and Vansteelandt (2009, 2014), VanderWeele (2016), and Jérolon *et al.* (2020).

Under the mediation model and with the above assumptions in place, we obtain

$$\text{NDE}=E\{Y(1,M(0))-Y(0,M(0))|X=x\}=\beta_{1},$$

and

$$\begin{array}{c} \text{NIE}=E\{Y(1,M(1))-Y(1,M(0))|X=x\}\\ =\sum_{j=1}^{m}\beta_{mj}\{\frac{\;\text{exp}(\alpha_{0j}+\alpha_{1j}+\alpha_{xj}^{⊤}x)}{1+\text{exp}(\gamma_{0j}+\gamma_{1j}+\gamma_{xj}^{⊤}x)}-\frac{\;\text{exp}(\alpha_{0j}+\alpha_{xj}^{⊤}x)}{1+\text{exp}(\gamma_{0j}+\gamma_{xj}^{⊤}x)}\}. \end{array}$$

The detailed derivations in this subsection are provided in the Supplementary Information.

Let *Y*($t,m_{j}$) denote the counterfactual value for *Y* if *T* were set to *t* and *M_j_* were set to *m_j_*. Define the path-specific mediation effect through *M_j_* as NIE${}_{j}=E\{Y$($1,M_{j}$(1))–*Y*($1,M_{j}$(0)) | X=x}. Then, under assumptions **A1–A4**, one can show that

$${\text{NIE}}_{j}=\beta_{mj}\{\frac{\;\text{exp}(\alpha_{0j}+\alpha_{1j}+\alpha_{xj}^{⊤}x)}{1+\text{exp}(\gamma_{0j}+\gamma_{1j}+\gamma_{xj}^{⊤}x)}-\frac{\;\text{exp}(\alpha_{0j}+\alpha_{xj}^{⊤}x)}{1+\text{exp}(\gamma_{0j}+\gamma_{xj}^{⊤}x)}\},$$

which implies that $\text{NIE}=\sum_{j=1}^{m}{\text{NIE}}_{j}$. NIE_*j*_ further can be decomposed into two distinct components. To see this, note that

$$M_{j}∼\{\begin{array}{cc} 0, & Z_{j}=1,\\ NB(\lambda_{ij},\phi_{j}), & Z_{j}=0, \end{array}$$

here *Z_j_* follows a Bernoulli distribution with parameter *π_j_*. By introducing the latent indicator *Z_j_*, we extend the path from $T\to M_{j}\to Y$ to $T\to Z_{j}\to M_{j}\to Y$ (Albert and Nelson 2011, Daniel *et al.* 2015). Let $Z_{j}$(*t*) denote the counterfactual value of *Z_j_* if exposure *T* were set to the value *t*. Similarly, $M_{j}$($t,z_{j}$) denotes the counterfactual value for *M_j_* if *T* were set to *t* and *Z_j_* were set to *z_j_*, and *Y*($t,z_{j},m_{j}$) denotes the counterfactual value for *Y* if *T* were set to *t*, *Z_j_* were set to *z_j_*, and *M_j_* were set to *m_j_*. With two ordered mediators, *Z_j_* and *M_j_*, an alternative expression for the path-specific mediation effect is NIE${}_{j}=E\{Y$($1,Z_{j}$(1)$,M_{j}$($1,Z_{j}$(1)))–*Y*($1,Z_{j}$(0)$,M_{j}$($0,Z_{j}$(0))) | X=x}. Consequently, NIE_*j*_ can be decomposed into two parts: one mediated by *Z_j_*,

$$\begin{array}{c} {\text{NIEP}}_{j}\\ =E\{Y(1,Z_{j}(1),M_{j}(1,Z_{j}(1)))-Y(1,Z_{j}(0),M_{j}(1,Z_{j}(0)))|X=x\}\\ =\beta_{mj}\left\{\frac{\;\text{exp}(\alpha_{0j}+\alpha_{1j}+\alpha_{xj}^{⊤}x)}{1+\text{exp}(\gamma_{0j}+\gamma_{1j}+\gamma_{xj}^{⊤}x)}-\frac{\;\text{exp}(\alpha_{0j}+\alpha_{1j}+\alpha_{xj}^{⊤}x)}{1+\text{exp}(\gamma_{0j}+\gamma_{xj}^{⊤}x)}\right\}, \end{array}$$

and the other by $M_{j}|Z_{j}=0$,

$$\begin{array}{c} {\text{NIEA}}_{j}\\ =E\{Y(1,Z_{j}(0),M_{j}(1,Z_{j}(0)))-Y(1,Z_{j}(0),M_{j}(0,Z_{j}(0)))|X=x\}\\ =\beta_{mj}\left\{\frac{\;\text{exp}(\alpha_{0j}+\alpha_{1j}+\alpha_{xj}^{⊤}x)}{1+\text{exp}(\gamma_{0j}+\gamma_{xj}^{⊤}x)}-\frac{\;\text{exp}(\alpha_{0j}+\alpha_{xj}^{⊤}x)}{1+\text{exp}(\gamma_{0j}+\gamma_{xj}^{⊤}x)}\right\}. \end{array}$$

In the context of microbiome data analysis, *Z_j_* serves as the presence–absence indicator for the *j*th microbe, and *M_j_* represents its abundance if present. Therefore, NIEP_*j*_ is the effect mediated by the presence status, while NIEA_*j*_ is the mediation effect through changes in the abundance.

### 2.3 Hypothesis testing of mediation effects

We are primarily concerned with the path-specific mediation effects. For the *j*th mediator, the null hypothesis of no natural indirect effect can be expressed as

$$H_{0j}:\beta_{mj}=0\;\text{or}\;\alpha_{1j}=\gamma_{1j}=0.$$

To test the first part of $H_{0j}$, say, whether $\beta_{mj}=0$, we adopt the distilled conditional randomization test (dCRT) introduced by Liu *et al.* (2022). This method, originally designed for normally distributed mediators, demonstrates robust control over the FDR in high-dimensional settings. To adapt dCRT for microbiome data, we modify it by substituting the normal distribution with the zero-inflated negative-binomial (ZINB) distribution. This adaptation yields the refined method referred to as zidCRT. Our proposed procedure of estimation and inference is outlined as follows:

Step 1. Run ZINB regression of $M_{j}=$(*M_ij_*) on ***T*** and X to obtain estimated parameters ${\hat{\pi }}_{j}=$(${\hat{\pi }}_{ij}$), ${\hat{\lambda }}_{j}=$(${\hat{\lambda }}_{ij}$), and ${\hat{\phi }}_{j}$. Denote ${\hat{M}}_{j}$ as the vector of fitted values;

Step 2. Fit LASSO regression of ***Y*** on ***T***, X, and $M_{-j}$ (all except the *j*th mediator), denoting ${\hat{Y}}_{j}$ the vector of fitted values;

Step 3. For b=1,…,B, sample $M_{j}^{\left(b\right)}=$($m_{ij}$)${}^{\left(b\right)}$ from the ZINB distribution with parameters ${\hat{\pi }}_{j}=$(${\hat{\pi }}_{ij}$), ${\hat{\lambda }}_{j}=$(${\hat{\lambda }}_{ij}$), and ${\hat{\phi }}_{j}$, and then repeat Step 1 to obtain ${\hat{M}}_{j}^{\left(b\right)}$;

Step 4. Calculate the *P*-value $p_{1j}$ as

$$\frac{1}{B+1}\left(1+\sum_{b=1}^{B}I\left\{\frac{\left|{\left(Y-{\hat{Y}}_{j}\right)}^{⊤}(M_{j}^{\left(b\right)}-{\hat{M}}_{j}^{\left(b\right)})\right|}{||M_{j}^{\left(b\right)}-{\hat{M}}_{j}^{\left(b\right)}|{|}^{2}}\geq \frac{\left|{\left(Y-{\hat{Y}}_{j}\right)}^{⊤}(M_{j}-{\hat{M}}_{j})\right|}{||M_{j}-{\hat{M}}_{j}|{|}^{2}}\right\}\right).$$

In our data analysis, we set *B *=* *100. In practice, a screening step may be added by applying LASSO regression of ***Y*** on ***T*** and **M**. Subsequently, Steps 1–4 are carried out conditional on the set D={*j*: the coefficient estimate of the *j*th mediator is nonzero}. All the tuning parameters are selected via cross-validation.

For the other part of $H_{0j}$, say, $\alpha_{1j}=\gamma_{1j}=0$, the Wald test is used. For any j∈D, let $\theta_{j}=$($\alpha_{1j},\gamma_{1j}$)${}^{⊤}$. The Wald-type statistic has the form

$$W^{2}={\hat{\theta }}_{j}{}^{⊤}{\left\{\hat{\mathrm{cov}}({\hat{\theta }}_{j})\right\}}^{-1}{\hat{\theta }}_{j},$$

where ${\hat{\theta }}_{j}$ is the maximum likelihood estimate of $\theta_{j}$ and $\hat{\mathrm{cov}}$(${\hat{\theta }}_{j}$) is the estimated asymptotic covariance matrix. Then we calculate the corresponding *P*-value, $p_{2j}$, based on an asymptotic chi-square distribution with 2 degrees of freedom.

Finally, the CRAmed method uses a joint significance test to determine whether to reject the null hypothesis, indicating no significant mediation effect of *M_j_* between treatment *T* and outcome *Y*. The *P*-value for the joint significance test is defined as $p_{\max}=\text{max}$($p_{1j},p_{2j}$). In this context, a microbe is considered a mediator only if both $p_{1j}$ and $p_{2j}$ are less than a chosen significance level.

## 3 Simulation study

In this section, we conducted a simulation study to investigate the effectiveness of the proposed method compared to existing methods, as summarized in Table 1. Note that some methods are tailored for normal mediators. To ensure a fair comparison, we log-transformed microbiome data after adding a pseudo-count of one to avoid logarithms for zeros, and we continued to refer to these methods as mentioned in Table 1.

**Table 1.** Summary of mediation analysis methods evaluated in the simulation study.

Method	Mediator type	Regularization technique
MultiMed (Boca *et al.* 2014)	Normal	No
HIMA (Zhang *et al.* 2016)	Normal/NB	SIS+MCP/SCAD/LASSO
HIMA2 (Perera *et al.* 2022)	Normal	SIS+De-biased LASSO
HDMA (Gao *et al.* 2019)	Normal	SIS+De-biased LASSO/Ridge
LDM-med (Yue and Hu 2022)	Count/binary	No
IKT (Imai *et al.* 2010)	Normal/Poisson	No
Naive-CRAmed	Normal	LASSO+dCRT
CRAmed	ZINB	LASSO+zidCRT

To assess the mediator selection performance of each method, we adjusted *P*-values by the Benjamini–Hochberg (BH) procedure. At the 5% FDR threshold, we calculated four metrics: the number of true positives (TP), representing the selected causal mediators; the number of false positives (FP), representing spurious mediators selected; the number of true negatives (TN), representing noncausal mediators correctly identified; and the number of false negatives (FN), representing causal mediators erroneously not selected. We then summarized these metrics into three criteria:

$$\text{Recall}=\frac{\text{TP}}{\text{TP}+\text{FN}},\text{Precision}=\frac{\text{TP}}{\text{TP}+\text{FP}},F1=2\times \frac{\text{Recall}\times \text{Precision}}{\text{Recall}+\text{Precision}},$$

all of which indicate better performance with larger values. We considered three simulation scenarios, with all results based on 100 data replications in each scenario.

### 3.1 Microbiome data generated from the ZINB model

We generated data from the mediation model described in Section 2. Specifically, we first generated microbiome data M= (*M_ij_*) using the following ZINB model:

$$M_{ij}∼\{\begin{array}{ccc} 0, & & \text{with}\;\text{probability}\;\pi_{ij},\\ NB(\lambda_{ij},\phi_{j}), & & \text{with}\;\text{probability}\;1-\pi_{ij}, \end{array}$$

with

$$\begin{array}{c} \text{logit}(\pi_{ij})=\gamma_{0j}+\gamma_{1j}T_{i},\\ \;\text{log}(\lambda_{ij})=\text{log}(S_{i})+\alpha_{0j}+\alpha_{1j}T_{i}, \end{array}$$

for i=1,…,n, j=1,…,m, where the treatment *T_i_* was sampled from a Bernoulli distribution with parameter 0.5, and the offset term log (*S_i_*), which adjusts for unequal sequencing depth, was sampled from *U*(7.1, 10.5), a uniform distribution on the interval (7.1, 10.5). Given the treatment *T_i_* and the mediators $M_{i}$, we generated the outcome *Y_i_* from a normal distribution with mean *μ_i_* and variance $\sigma^{2}$, where $\mu_{i}=\beta_{0}+\beta_{1}T_{i}+\beta_{m}^{⊤}M_{i}$.

Details on the regression coefficients are listed in Table 2. Note that only the first 5 taxa are considered causal, and the proportion of zeros in the data matrix **M** is approximately 50%–60%. To investigate the effects of sample size (*n*) and the dimension of mediators (*m*) on the performance, we considered four different combinations of (*n*, *m*) with n∈{100,200} and m∈{100,1000}.

**Table 2.** Specification of parameters in the mediation model examined in Section 2.^a^

Taxon index (*j*)	$\gamma_{1j}$	$\alpha_{1j}$	*β_mj_*
1, 2, 3	N(−2,1)	N(−2,1)	*N*(2, 1)
4	0	N(−2,1)	*N*(2, 1)
5	N(−2,1)	0	*N*(2, 1)
6,…,10	0	0	*N*(2, 1)
11,…,15	N(−2,1)	N(−2,1)	0
16,…,20	N(−2,1)	0	0
21,…,25	0	N(−2,1)	0
26,…,m	0	0	0

a We sampled $\phi_{j}$ from $U(0.1,10),\;\gamma_{0j}$ from U(−2,2), and set $\alpha_{0j}=-7,\;\beta_{0}=1,\;\beta_{1}=-2$, and $\sigma^{2}=1$.

**Figure 1.**
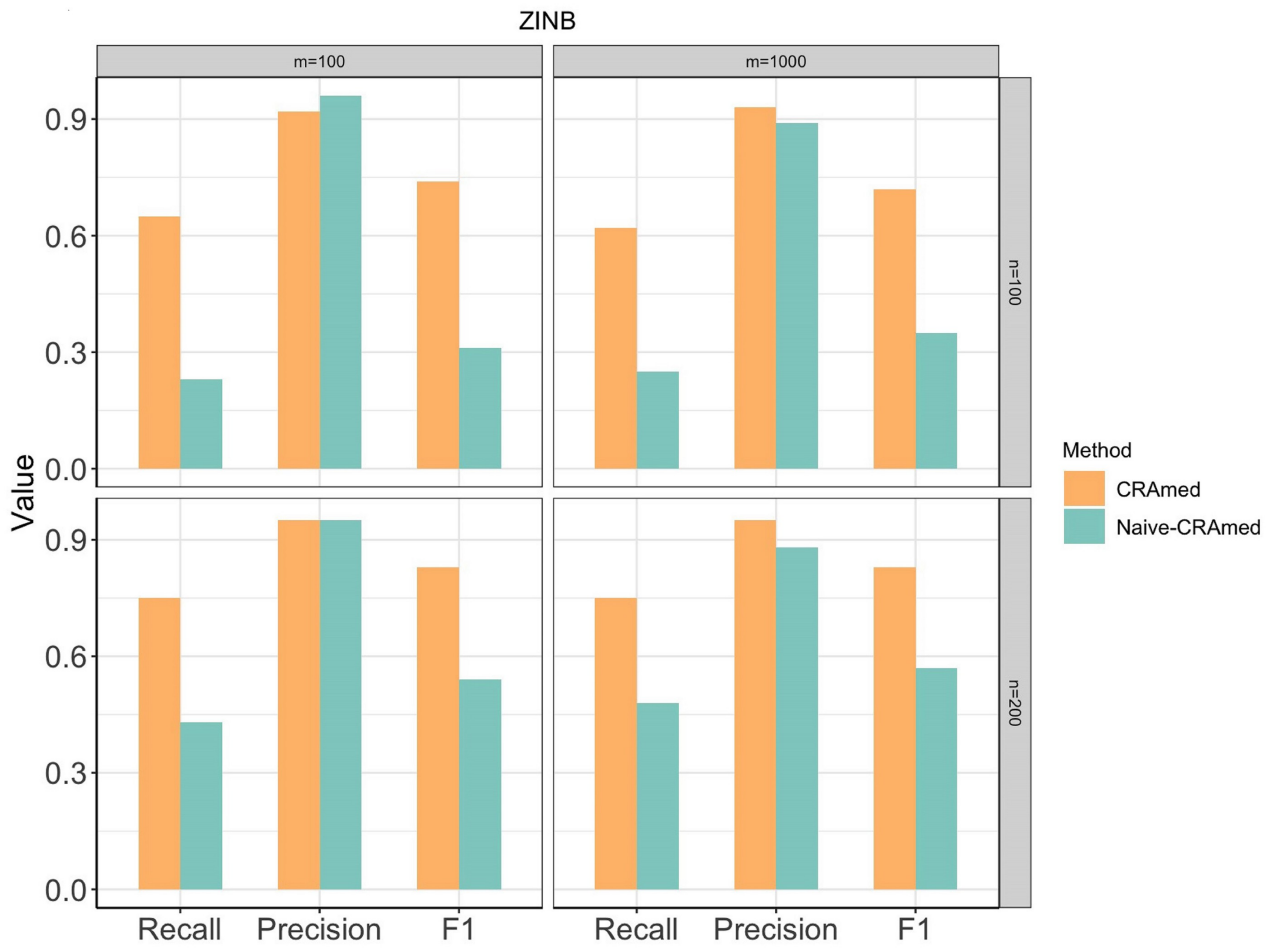
Comparison of Recall, Precision, and F1 score for the Naive-CRAmed and CRAmed methods using microbiome data generated from the ZINB model. Sample size n∈{100,200} and number of taxa m∈{100,1000}.

**Figure 2.**
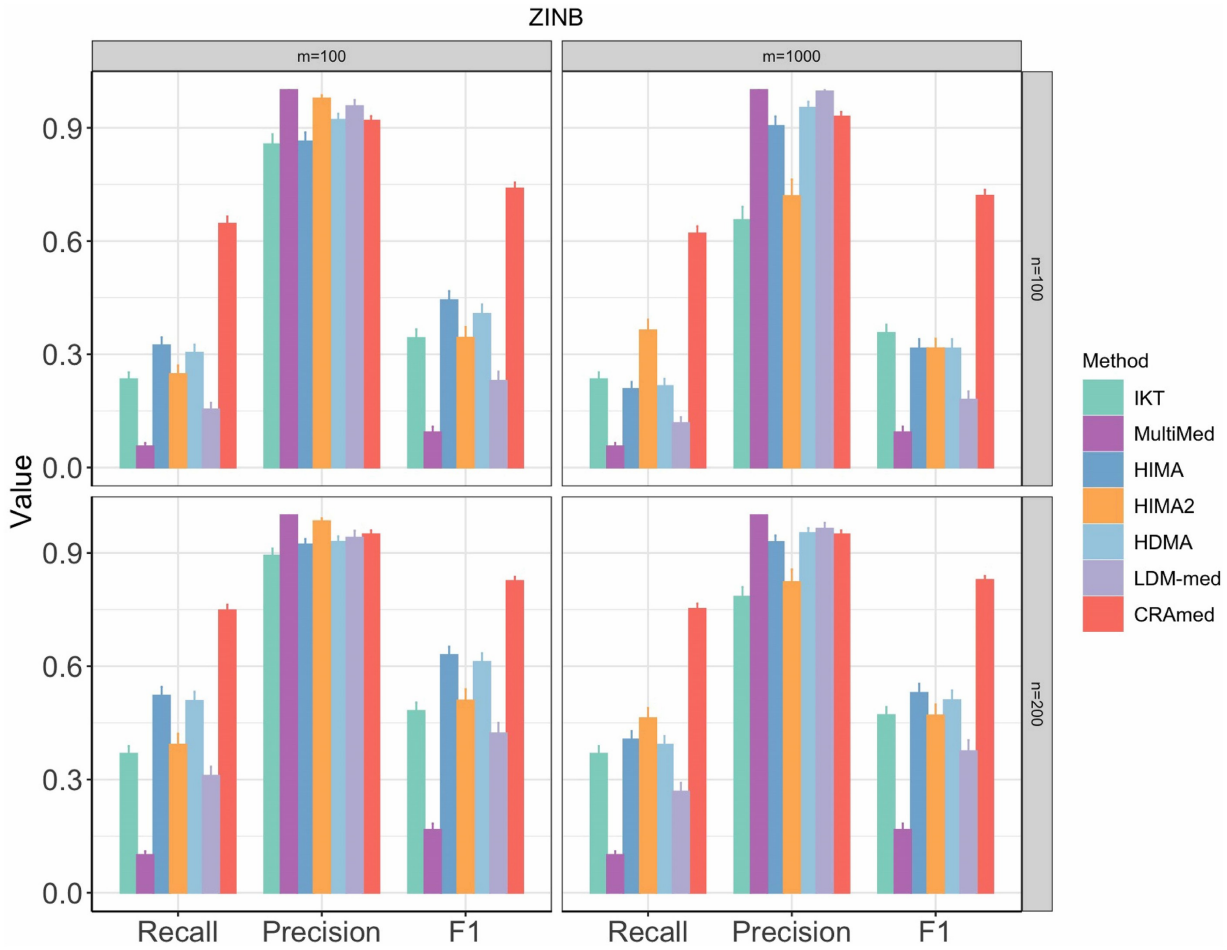
Comparison of Recall, Precision, and F1 score for different mediation analysis methods using microbiome data generated from the ZINB model. Sample size n∈{100,200} and number of taxa m∈{100,1000}.

The main difference between Naive-CRAmed and CRAmed lies in their approach to evaluating mediation effects. While the former utilizes the dCRT method for normally distributed mediators, the latter uses the proposed zidCRT method, specifically designed to handle zero-inflated mediators. Figure 1 illustrates that both Naive-CRAmed and CRAmed attain high precision when the number of taxa *m *=* *100. However, Naive-CRAmed exhibits significantly lower Recall, mainly because it fails to address the inherent sparsity in microbiome data. Figure 2 compares the performance of CRAmed with that of MultiMed, HIMA, HIMA2, HDMA, LDM-med, and IKT. It is evident that CRAmed consistently achieves the highest Recall and F1 score while maintaining competitively high precision across all settings. This suggests that CRAmed possesses the ability to more effectively select true causal mediators while excluding nonmediators. While HIMA2 is specifically designed to handle high-dimensional mediators, it shows poor precision when the number of taxa *m *=* *1000. The MultiMed method performs best in terms of precision, but it exhibits the smallest Recall and F1 score. The superiority of CRAmed over its competitors stems from its ability to handle both zero-inflation and over-dispersion without requiring data transformations to conform to normality.

### 3.2 Microbiome data generated from hurdle Poisson and hurdle NB models

To evaluate the robustness of the proposed CRAmed method against violations of the ZINB model assumption, we generated microbiome data from the hurdle Poisson and hurdle NB models. The probability function of hurdle NB model is defined as follows:

$$\begin{array}{c} P(M_{ij}=0)=\pi_{ij},\\ P(M_{ij}=k)=\frac{\left(1-\pi_{ij}\right)}{1-{\left(\frac{\phi_{j}}{\phi_{j}+\lambda_{ij}}\right)}^{\phi_{j}}}\frac{\Gamma (\phi_{j}+k)}{k!\Gamma (\phi_{j})}{\left(\frac{\phi_{j}}{\phi_{j}+\lambda_{ij}}\right)}^{\phi_{j}}\\ \times {\left(\frac{\lambda_{ij}}{\phi_{j}+\lambda_{ij}}\right)}^{k},k=1,2,3,\ldots \end{array}$$

with

$$\begin{array}{c} \text{logit}(\pi_{ij})=\gamma_{0j}+\gamma_{1j}T_{i},\\ \;\text{log}(\lambda_{ij})=\text{log}(S_{i})+\alpha_{0j}+\alpha_{1j}T_{i}, \end{array}$$

for i=1,…,n, j=1,…,m. Setting $\phi_{j}=\infty$ reduces the hurdle NB model to the hurdle Poisson model. By definition, the probabilities for positive counts are determined by the NB or Poisson distribution truncated at zero.

The rest of simulation settings are the same as those of the previous example, and the results are summarized in Supplementary Figs S1–S4 in the Supplementary Information. The conclusions remain qualitatively unchanged. Despite model misspecification, the proposed CRAmed method still outperforms others in identifying causal taxa, underscoring its robustness.

### 3.3 Microbiome data generated with unobserved confounders

So far, microbiome data were generated under the assumption of no unmeasured confounding. To comprehensively evaluate the robustness and reliability of CRAmed and its competitors, we have expanded the simulation study to include a broader range of settings for generating unmeasured confounders, systematically violating each of the A1–A4 assumptions to varying degrees. Specifically, to violate A1 assumption, we generated the treatment *T_i_* from a Bernoulli distribution with parameter *η_ij_*, where

$$\text{logit}(\eta_{ij})=\Delta_{uj}^{⊤}X_{i}^{u}.$$

Here, $X_{i}^{u}$ denotes a *d*-vector of unobserved confounders. The microbiome data were generated from the ZINB model. Given the treatment *T_i_* and the mediators $M_{i}$, we generated the outcome *Y_i_* from a normal distribution $N(\mu_{i},\sigma^{2})$, where

$$\mu_{i}=\beta_{0}+\beta_{1}T_{i}+\beta_{m}^{⊤}M_{i}+\beta_{u}^{⊤}X_{i}^{u}.$$

With *d *=* *3, the unobserved confounders were sampled independently from *N*(0, 1). To evaluate how these confounders affect the performance of various methods, we sampled $\Delta_{uj}$ and $\beta_{u}$ independently from N(0.2h,0.5), where h∈{1,3,9}. The remaining settings were consistent with the previous examples, except that the regression coefficients of $\gamma_{1},\;\alpha_{1}$, and those of $\beta_{m}$, were drawn from N(−5,1) and *N*(5, 1), respectively.

**Figure 3.**
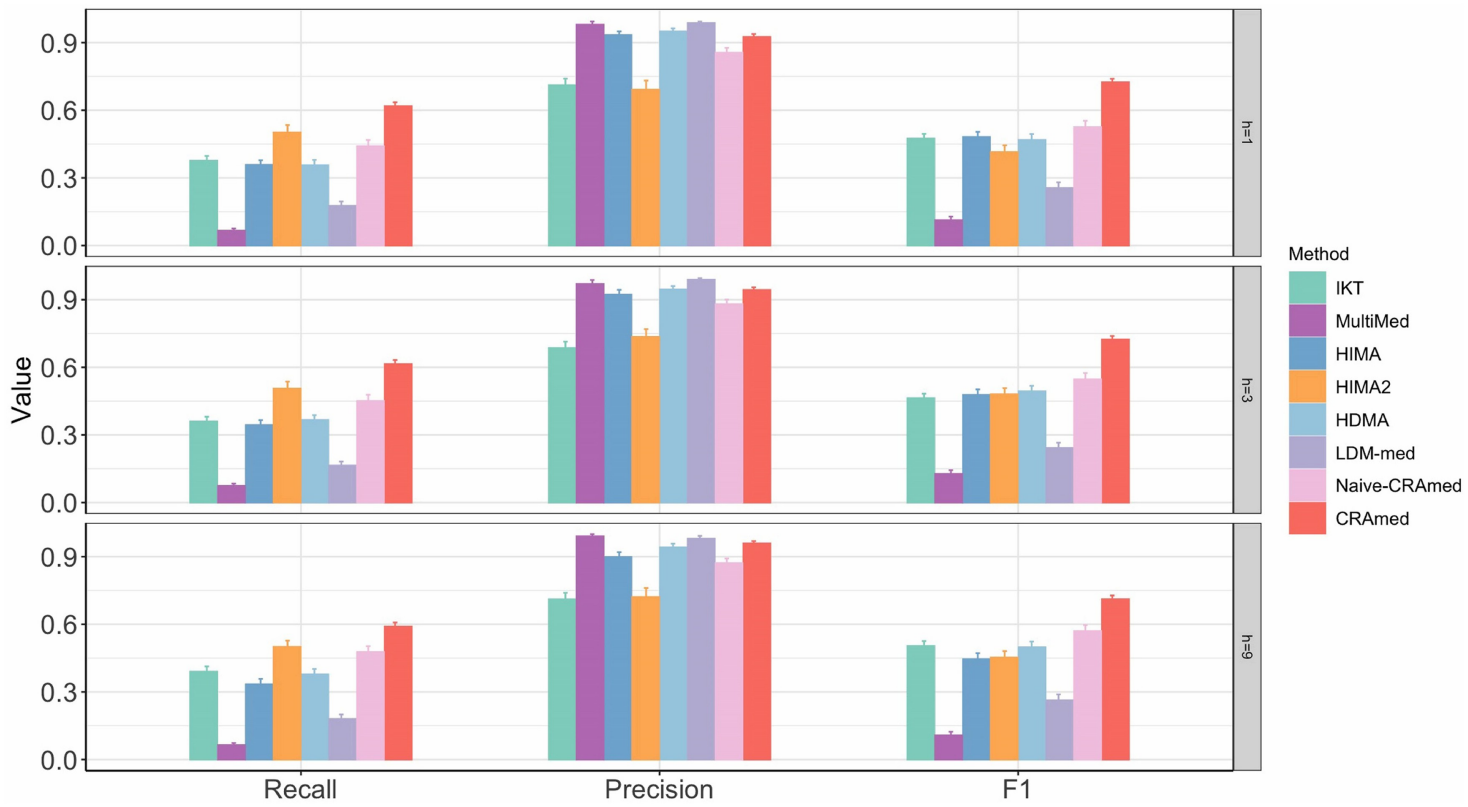
Comparison of Recall, Precision, and F1 score in the sensitivity analysis using microbiome data generated from the ZINB model, with unobserved confounders present in the relationship between treatment and outcome. Sample size *n *=* *100 and number of taxa *m *=* *1000.

Simulation results are shown in Fig. 3 for (*n*, *m*)= (100, 1000), and in Supplementary Figs S5–S19 for other scenarios. When unmeasured confounders influenced the relationship between treatment and outcome, we observe that the performance of all methods deteriorated as the extent of violation (*h*) increased. Nevertheless, CRAmed consistently achieves the highest F1 score while maintaining competitively high precision across all settings, highlighting the robustness of CRAmed in the presence of unobserved confounders between treatment and outcome. Nevertheless, extending CRAmed while accounting for unmeasured confounding is important, although this is beyond the scope of the present article.

Finally, we conducted a simulation study comparing the computational time of CRAmed with that of other methods. We observed from Supplementary Fig. S20 that CRAmed’s computational time scales linearly with the number of taxa.

## 4 Real data applications

### 4.1 Identification of microbial mediators of weight under different modes of delivery

Delivery mode affects stability of early infant gut microbiota (Mitchell *et al.* 2020). Several studies have demonstrated that cesarean section (C-section) disrupts the succession of the newborn microbiome originating from the maternal birth canal, thereby increasing the risk of adverse health outcomes in offspring compared with vaginally delivered infants (Andersen *et al.* 2020, Korpela *et al.* 2020, Zhou *et al.* 2023). Consequently, it is crucial to understand how the delivery mode influences the infant gut microbiome, subsequently mediating the phenotype of infants.

**Figure 4.**
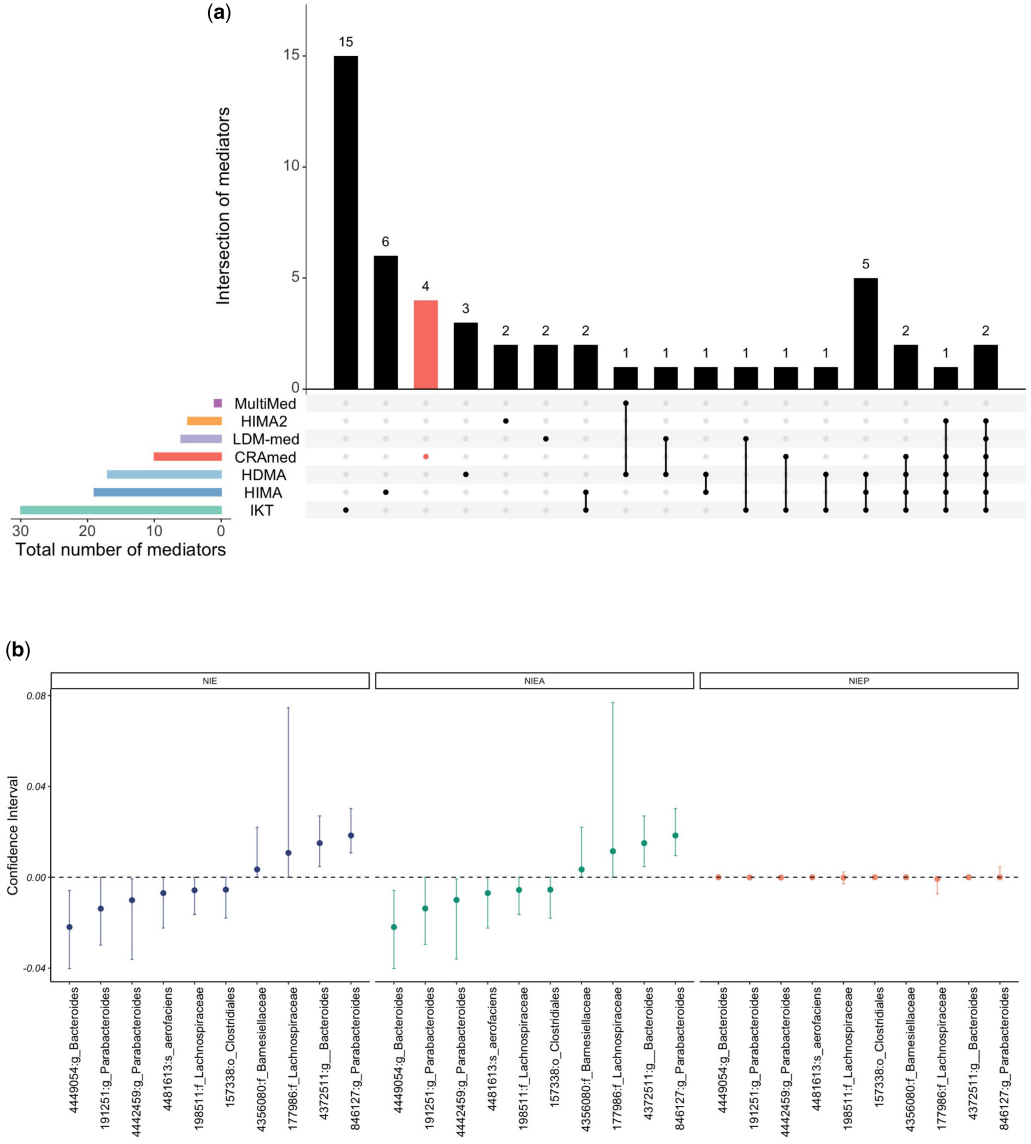
Mediation analyses for the DIABIUMMUNE dataset. (a) UpSetR plot illustrates the number of mediators shared between different mediation analysis methods. (b) Point and 95% CI estimates of NIE, NIEA, and NIEP for CRAmed-identified OTUs mediating the effect of modes of delivery on the weights. The 95% CI estimates were calculated based on the permutation strategy with 1000 repetitions.

We analyzed a publicly available gut microbiome dataset from a previous study (Yassour *et al.* 2016), which includes clinical examinations and gut microbiome data from 1098 infants. Our investigation focused on the outcome variable of weight growth pace during the first year, aiming to explore whether the gut microbiome mediates the relationship between delivery mode and the infants’ weight. After filtering out taxa with a prevalence of <10%, we obtained a dataset consisting of 876 taxa and 1098 samples. The results at the 5% FDR level are shown in Fig. 4a. CRAmed detected 10 operational taxonomic units (OTUs), a moderate number of mediators, significantly mediating the relationship between delivery mode and BMI. Furthermore, CRAmed was less conservative than Naive-CRAmed, which identified no mediators, and MultiMed, which identified only one mediator. Yet, it is not as liberal as IKT, HIMA, and HDMA, which tended to produce an excess of false positives, as indicated in previous simulation studies.

Upon closer examination of the 10 mediators identified by CRAmed, they are affiliated with three genera: *Bacteroides*, *Parabacteroides*, and *Collinsella*. Previous investigations have established that all three genera are more prevalent in the vaginal delivery group and are associated with both the mode of delivery and weight gain (Arboleya *et al.* 2017, Li *et al.* 2021, Wang *et al.* 2021b; Mancabelli *et al.* 2023).

As described in Section 2, the natural indirect effect (NIE) of a taxon can be broken down into two components: the effect mediated by the presence status (NIEP) and the effect through changes in the abundance level (NIEA). To investigate these effects, we constructed 95% confidence intervals for each of the 10 OTUs identified by CRAmed. This was achieved using a permutation-based approach, involving 1000 random shufflings of the data. Taking the identified mediator OTU 846127, affiliated with the genus *Parabacteroides*, as an illustrative example, Fig. 4b demonstrates that, rather than the presence of this taxon, its abundance plays a pivotal role in mediating the relationship between C-sections and weight loss.

### 4.2 Identification of microbial mediators of BMI and waist circumference under antibiotic treatment

Numerous studies have highlighted the significant impact of antibiotics on the composition of gut microbiota (Cho *et al.* 2012, Fishbein *et al.* 2023). In this section, we extend the application of CRAmed to investigate the interplay among antibiotic intake, gut microbiome, and risk factors associated with cardiometabolic diseases (CMD). These risk factors include body mass index (BMI), waist circumference (WC), high density lipoprotein (HDL), low density lipoprotein (LDL), total cholesterol (TCHO), triglycerides (TG), fasting blood glucose (FBG), systolic blood pressure (SBP), and diastolic blood pressure (DBP). We utilized a dataset from the Guangdong Gut Microbiome Project (GGMP), a large community-based cross-sectional cohort conducted between 2015 and 2016 (He *et al.* 2018). Before proceeding, we filtered taxa with prevalence <10%, leaving 944 taxa in 894 samples. Detailed information on data pre-processing and preliminary results of exploratory data analysis are available in the Supplementary Information.

**Figure 5.**
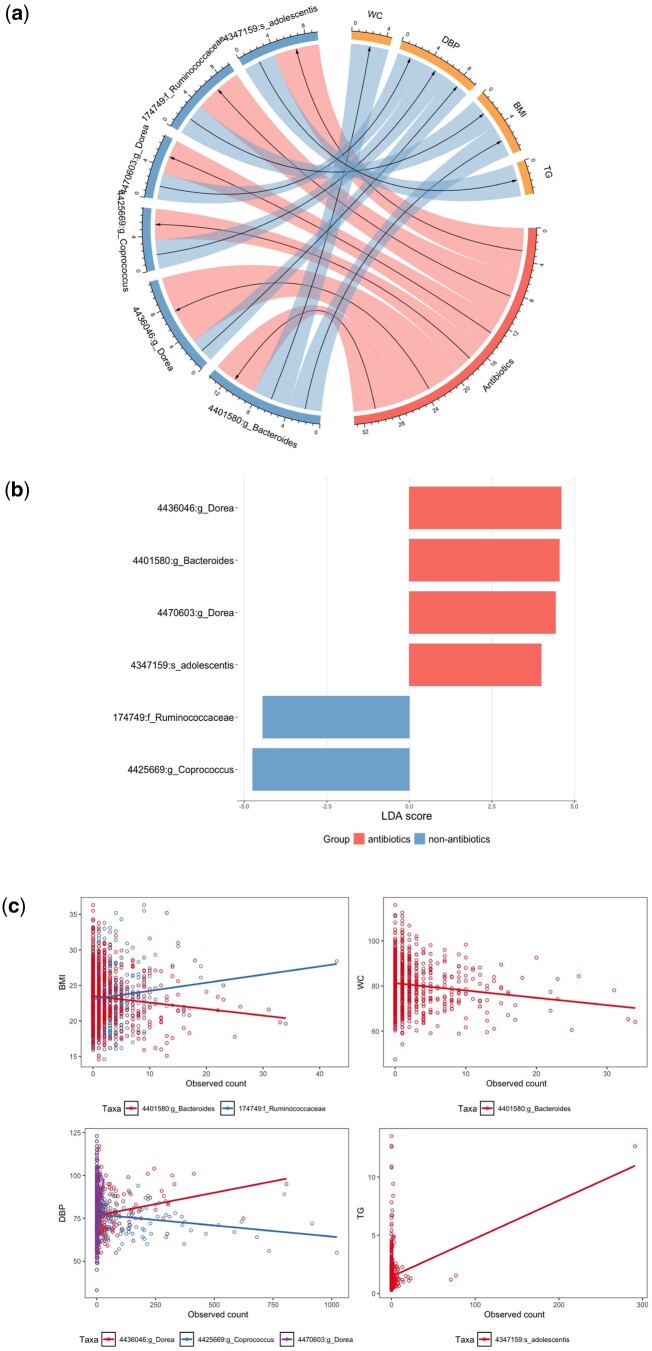
Mediation analyses for the GGMP dataset. (a) Associations between antibiotic treatment and gut microbiota, as well as between gut microbiota and CMD-related risk factors (BMI, WC, TG, and DBP). The width of each strip is depicted by −log (*P*-value). (b) LDA score representing the differences of the OTUs identified by CRAmed between the antibiotics and non-antibiotics groups. Taxa with LDA scores greater than 0 are enriched in the antibiotics group, while those with LDA scores less than 0 are enriched in the non-antibiotics group. (c) Scatterplots of CMD-related risk factors and the relative abundances of the identified mediators.

CRAmed identified six causal microbial taxa that significantly mediated the relationship between antibiotic treatment and CMD-related risk factors, as illustrated in Fig. 5a. Notably, the OTU 4401580 mediates the connection between antibiotic intake and both BMI and waist circumference, and the OTU 174749 shows significance in mediating the association between antibiotic intake and BMI. From Fig. 5b and c, it is evident that the OTU 4401580, affiliated with the genus *Bacteroides*, undergoes a significant increase following antibiotics treatment, whereas the OTU 174749, affiliated with the family *Ruminococcaceae*, displays the opposite trend. Furthermore, a strong negative correlation between the OTU 4401580 and BMI can be observed. Using a murine model of high-fat diet-induced obesity, Liu *et al.* (2019) investigated the impact of antibiotics-induced gut microbial perturbations on metabolic phenotypes. They reported that the relative abundance of *Bacteroides* increased to more than twice the original levels after antibiotics treatment, while *Ruminococcaceae* showed a decrease compared to baseline levels. Additionally, they highlighted a negative correlation between the abundance of *Bacteroidetes* and body weight gain. For the family *Ruminococcaceae*, it was also reported to have the strongest positive correlation with body weight (Nemoto *et al.* 2023).

Permutation-based 95% confidence intervals of NIE, NIEA, and NIEP for each of the identified taxa are depicted in Supplementary Fig. S10. It is evident that both the OTU 4401580 and the OTU 174749 exhibit positive mediation effects on BMI, and the former also shows a positive mediation effect on WC. Furthermore, for both taxa, it is the abundance level, rather than the presence status, that plays a role in mediating the relationship between antibiotic treatment and BMI or WC.

## 5 Discussion

The human microbiome is a key determinant of normal physiology and immune homeostasis, providing essential functions such as immune system regulation, metabolic processes, and vitamin synthesis (Honda and Littman 2016, Thaiss *et al.* 2016, Heintz-Buschart and Wilmes 2018). Additionally, the microbiome has been shown to change readily in response to extrinsic factors such as diet and xenobiotics (Wu *et al.* 2011, Lewis *et al.* 2015, Kurilshikov *et al.* 2017). Therefore, understanding the mechanisms underlying the effects of external factors or interventions on diseases transmitted through microbiome perturbations is crucial. Although standard mediation analysis methods are widely used in this context, model specification and statistical inference require careful consideration of the unique characteristics inherent in microbiome data.

In this article, we have proposed CRAmed for decomposing and testing the mediation effects of the microbiome in the relationship between a treatment and an outcome. To address the characteristics of microbiome data, CRAmed utilizes the ZINB model and introduces a conditional randomization test, zidCRT. By introducing the latent indicator for the presence–absence status of a taxon, CRAmed decomposes the natural indirect effect into two components, thereby enhancing the interpretability of mediation analysis.

Comprehensive simulations have demonstrated the superior performance of CRAmed in Recall, precision, and F1 score, with a notable level of robustness, compared to existing mediation analysis methods. Furthermore, two real data applications have illustrated the effectiveness and interpretability of CRAmed. These findings suggest that CRAmed holds promise for investigating causal microbes and gaining a better understanding of the factors influencing host health.

One limitation inherent in our method, akin to other statistical mediation models, is its reliance on several assumptions, including the absence of unmeasured confounders. It adheres to the fundamental principle that association does not inherently imply causality, as emphasized by MacKinnon and Fairchild (2009) and VanderWeele and Vansteelandt (2009). Moreover, in scenarios involving multiple outcomes and high-dimensional mediators, CRAmed faces significant computational challenges due to its reliance on empirical *P*-values, which require extensive permutation testing. Addressing these complexities represents an intriguing direction for future research.

## Author contributions

T.W. and P.R.X. oversaw the study. The theory underlying CRAmed was conceived of and developed by T.T.L., with contributions from X.N.X. T.T.L. performed simulation studies, and real data analyses and developed the CRAmed R package. T.T.L. wrote the first version of the manuscript. X.N.X., T.W., and P.R.X. also contributed to the writing. The authors read and approved the final manuscript.

## Supplementary Material

btaf038_Supplementary_Data

## Data Availability

The two real microbiome datasets are available at the R package CRAmed. The proposed method is implemented in the R package CRAmed, publicly available at Github: https://github.com/liudoubletian/CRAmed.
