# Supplementary material for: CRAmed: a conditional randomization test for high-dimensional mediation analysis in sparse microbiome data
Source: Bioinformatics. 2025 Jan 28;41(2):btaf038. doi: 10.1093/bioinformatics/btaf038 (PMC11821267; doi:10.1093/bioinformatics/btaf038)
Supplement: btaf038_Supplementary_Data [file btaf038_supplementary_data.zip › 6a91d_CRAmed_supplementary_final.pdf]

Supplementary information for

CRAmed: A conditional randomization test for high-dimensional mediation analysis in sparse microbiome data

Tiantian Liu[1], Xiangnan Xu[2], Tao Wang[3,4,5,6,*], and Peirong Xu[4,*]

[1]Research Center of Biostatistics and Computational Pharmacy, China Pharmaceutical University, 639 Longmian Dadao, Nanjing, 211198, China
[2]Chair of Statistics, Humboldt-Universität zu Berlin, Unter den Linden 6, Berlin, 10099, Germany
[3]SJTU-Yale Joint Center of Biostatistics and Data Science, Shanghai Jiao Tong University, 800 Dongchuan RD, Shanghai, 200240, China
[4]Department of Statistics, School of Mathematical Sciences, Shanghai Jiao Tong University, 800 Dongchuan RD, Shanghai, 200240, China
[5]MOE-LSC & CMA-Shanghai, Shanghai Jiao Tong University, 800 Dongchuan RD, Shanghai, 200240, China
[6]MoE Key Lab of Artificial Intelligence, AI Institute, Shanghai Jiao Tong University, 800 Dongchuan RD, Shanghai, 200240, China
[*]Corresponding author: neowangtao@sjtu.edu.cn; prxu@sjtu.edu.cn

Contents

I Technical details 3

I-A Derivation of the average natural direct effect and average natural indirect effect 3

I-B Decomposition for the average natural indirect effect 4

II Additional simulation studies 7

III Preprocessing and additional results for the application studies in Section 3 21

III-A Identification of microbial mediators of weight under different modes of delivery 21

III-B Identification of microbial mediators of BMI and waist circumference under antibiotic treatment 21

I. Technical details

A. Derivation of the average natural direct effect and average natural indirect effect

Using the notation in the main text, we can derive the average natural direct effect (NDE) and average natural indirect effect (NIE) as follows:

$$
\begin{aligned}
\text{NDE} &= E\{Y(1,\boldsymbol{M}(0)) - Y(0,\boldsymbol{M}(0)) \mid \boldsymbol{X}=\boldsymbol{x}\} \\
&= E\{Y(1,\boldsymbol{M}(0)) \mid \boldsymbol{X}=\boldsymbol{x}\} - E\{Y(0,\boldsymbol{M}(0)) \mid \boldsymbol{X}=\boldsymbol{x}\} \\
&= \sum_{m} E\{Y(1,\boldsymbol{m}) \mid \boldsymbol{M}(0)=\boldsymbol{m},\boldsymbol{x}\} P(\boldsymbol{M}(0)=\boldsymbol{m} \mid \boldsymbol{x}) \\
&\quad - \sum_{m} E\{Y(0,\boldsymbol{m}) \mid \boldsymbol{M}(0)=\boldsymbol{m},\boldsymbol{x}\} P(\boldsymbol{M}(0)=\boldsymbol{m} \mid \boldsymbol{x}) \\
&\overset{\textbf{A4}}{=} \sum_{m} E\{Y(1,\boldsymbol{m}) \mid \boldsymbol{X}=\boldsymbol{x}\} P(\boldsymbol{M}(0)=\boldsymbol{m} \mid \boldsymbol{X}=\boldsymbol{x}) \\
&\quad - \sum_{m} E\{Y(0,\boldsymbol{m}) \mid \boldsymbol{X}=\boldsymbol{x}\} P(\boldsymbol{M}(0)=\boldsymbol{m} \mid \boldsymbol{X}=\boldsymbol{x}) \\
&\overset{\textbf{A1}}{=} \sum_{m} E\{Y(1,\boldsymbol{m}) \mid T=1,\boldsymbol{X}=\boldsymbol{x}\} P(\boldsymbol{M}(0)=\boldsymbol{m} \mid \boldsymbol{X}=\boldsymbol{x}) \\
&\quad - \sum_{m} E\{Y(0,\boldsymbol{m}) \mid T=0,\boldsymbol{X}=\boldsymbol{x}\} P(\boldsymbol{M}(0)=\boldsymbol{m} \mid \boldsymbol{X}=\boldsymbol{x}) \\
&\overset{\textbf{A2}}{=} \sum_{m} E\{Y(1,\boldsymbol{m}) \mid T=1,\boldsymbol{M}=\boldsymbol{m},\boldsymbol{X}=\boldsymbol{x}\} P(\boldsymbol{M}(0)=\boldsymbol{m} \mid \boldsymbol{X}=\boldsymbol{x}) \\
&\quad - \sum_{m} E\{Y(0,\boldsymbol{m}) \mid T=0,\boldsymbol{M}=\boldsymbol{m},\boldsymbol{X}=\boldsymbol{x}\} P(\boldsymbol{M}(0)=\boldsymbol{m} \mid \boldsymbol{X}=\boldsymbol{x}) \\
&\overset{\textbf{A3}}{=} \sum_{m} E\{Y(1,\boldsymbol{m}) \mid T=1,\boldsymbol{M}=\boldsymbol{m},\boldsymbol{X}=\boldsymbol{x}\} P(\boldsymbol{M}(0)=\boldsymbol{m} \mid T=0,\boldsymbol{X}=\boldsymbol{x}) \\
&\quad - \sum_{m} E\{Y(0,\boldsymbol{m}) \mid T=0,\boldsymbol{M}=\boldsymbol{m},\boldsymbol{X}=\boldsymbol{x}\} P(\boldsymbol{M}(0)=\boldsymbol{m} \mid T=0,\boldsymbol{X}=\boldsymbol{x}) \\
&= \sum_{m} E\{Y \mid T=1,\boldsymbol{M}=\boldsymbol{m},\boldsymbol{X}=\boldsymbol{x}\} P(\boldsymbol{M}(0)=\boldsymbol{m} \mid T=0,\boldsymbol{X}=\boldsymbol{x}) \\
&\quad - \sum_{m} E\{Y \mid T=0,\boldsymbol{M}=\boldsymbol{m},\boldsymbol{X}=\boldsymbol{x}\} P(\boldsymbol{M}(0)=\boldsymbol{m} \mid T=0,\boldsymbol{X}=\boldsymbol{x}) \\
&= \left[\beta_0 + \beta_1 + \sum_{j=1}^{p} \beta_{mj} E\{M_j \mid T=0,\boldsymbol{X}=\boldsymbol{x}\} + \boldsymbol{\beta}_x^\top \mathbf{X}\right] - \left[\beta_0 + \sum_{j=1}^{m} \beta_{mj} E\{M_j \mid T=0,\boldsymbol{X}=\boldsymbol{x}\} + \boldsymbol{\beta}_x^\top \mathbf{x}\right] \\
&= \beta_1.
\end{aligned}
$$

$$
\begin{aligned}
\text{NIE} &= E\{Y(1,\boldsymbol{M}(1)) - Y(1,\boldsymbol{M}(0)) \mid \boldsymbol{X}=\boldsymbol{x}\} \\
&= E\{Y(1,\boldsymbol{M}(1)) \mid \boldsymbol{X}=\boldsymbol{x}\} - E\{Y(1,\boldsymbol{M}(0)) \mid \boldsymbol{X}=\boldsymbol{x}\} \\
&= \sum_{m} E\{Y(1,\boldsymbol{m}) \mid \boldsymbol{M}(1)=\boldsymbol{m}, \boldsymbol{X}=\boldsymbol{x}\} P(\boldsymbol{M}(1)=\boldsymbol{m} \mid \boldsymbol{X}=\boldsymbol{x}) \\
&\quad - \sum_{m} E\{Y(1,\boldsymbol{m}) \mid \boldsymbol{M}(0)=\boldsymbol{m}, \boldsymbol{X}=\boldsymbol{x}\} P(\boldsymbol{M}(0)=\boldsymbol{m} \mid \boldsymbol{X}=\boldsymbol{x}) \\
&\overset{\textbf{A4}}{=} \sum_{m} E\{Y(1,\boldsymbol{m}) \mid \boldsymbol{X}=\boldsymbol{x}\} P(\boldsymbol{M}(1)=\boldsymbol{m} \mid \boldsymbol{X}=\boldsymbol{x}) \\
&\quad - \sum_{m} E\{Y(1,\boldsymbol{m}) \mid \boldsymbol{X}=\boldsymbol{x}\} P(\boldsymbol{M}(0)=\boldsymbol{m} \mid \boldsymbol{X}=\boldsymbol{x}) \\
&\overset{\textbf{A1}}{=} \sum_{m} E\{Y(1,\boldsymbol{m}) \mid T=1, \boldsymbol{X}=\boldsymbol{x}\} P(\boldsymbol{M}(1)=\boldsymbol{m} \mid \boldsymbol{X}=\boldsymbol{x}) \\
&\quad - \sum_{m} E\{Y(1,\boldsymbol{m}) \mid T=1, \boldsymbol{X}=\boldsymbol{x}\} P(\boldsymbol{M}(0)=\boldsymbol{m} \mid \boldsymbol{X}=\boldsymbol{x}) \\
&\overset{\textbf{A2}}{=} \sum_{m} E\{Y(1,\boldsymbol{m}) \mid T=1, \boldsymbol{M}=\boldsymbol{m}, \boldsymbol{X}=\boldsymbol{x}\} P(\boldsymbol{M}(1)=\boldsymbol{m} \mid \boldsymbol{X}=\boldsymbol{x}) \\
&\quad - \sum_{m} E\{Y(1,\boldsymbol{m}) \mid T=1, \boldsymbol{M}=\boldsymbol{m}, \boldsymbol{X}=\boldsymbol{x}\} P(\boldsymbol{M}(0)=\boldsymbol{m} \mid \boldsymbol{X}=\boldsymbol{x}) \\
&\overset{\textbf{A3}}{=} \sum_{m} E\{Y(1,\boldsymbol{m}) \mid T=1, \boldsymbol{M}=\boldsymbol{m}, \boldsymbol{X}=\boldsymbol{x}\} P(\boldsymbol{M}(1)=\boldsymbol{m} \mid T=1, \boldsymbol{X}=\boldsymbol{x}) \\
&\quad - \sum_{m} E\{Y(1,\boldsymbol{m}) \mid T=1, \boldsymbol{M}=\boldsymbol{m}, \boldsymbol{X}=\boldsymbol{x}\} P(\boldsymbol{M}(0)=\boldsymbol{m} \mid T=0, \boldsymbol{X}=\boldsymbol{x}) \\
&= \sum_{m} E\{Y \mid T=1, \boldsymbol{M}=\boldsymbol{m}, \boldsymbol{X}=\boldsymbol{x}\} P(\boldsymbol{M}(1)=\boldsymbol{m} \mid T=1, \boldsymbol{X}=\boldsymbol{x}) \\
&\quad - \sum_{m} E\{Y \mid T=1, \boldsymbol{M}=\boldsymbol{m}, \boldsymbol{X}=\boldsymbol{x}\} P(\boldsymbol{M}(0)=\boldsymbol{m} \mid T=0, \boldsymbol{X}=\boldsymbol{x}) \\
&= \left(\beta_0 + \beta_1 + \sum_{j=1}^{m} \beta_{mj} E\{M_j \mid T=1, \boldsymbol{X}=\boldsymbol{x}\} + \boldsymbol{\beta}_x^\top \boldsymbol{x}\right) \\
&\quad - \left(\beta_0 + \beta_1 + \sum_{j=1}^{m} \beta_{mj} E\{M_j \mid T=0, \boldsymbol{X}=\boldsymbol{x}\} + \boldsymbol{\beta}_x^\top \boldsymbol{x}\right) \\
&= \sum_{j=1}^{m} \beta_{mj} \times \left[E\{M_j \mid T=1, \boldsymbol{X}=\boldsymbol{x}\} - E\{M_j \mid T=0, \boldsymbol{X}=\boldsymbol{x}\}\right] \\
&= \sum_{j=1}^{m} \beta_{mj} \times \left\{ \frac{\exp(\alpha_{0j} + \alpha_{1j} + \boldsymbol{\alpha}_{xj}^\top \boldsymbol{x})}{1+\exp(\gamma_{0j} + \gamma_{1j} + \boldsymbol{\gamma}_{xj}^\top \boldsymbol{x})} - \frac{\exp(\alpha_{0j} + \boldsymbol{\alpha}_{xj}^\top \boldsymbol{x})}{1+\exp(\gamma_{0j} + \boldsymbol{\gamma}_{xj}^\top \boldsymbol{x})} \right\}.
\end{aligned}
$$

B. Decomposition for the average natural indirect effect

To identify the path specific effects NIEP_j and NIEA_j mentioned in main text, we extend the preceding assumptions **A1-A4** to the two ordered mediators model case. And the assumptions is given as:
B1: No unmeasured confounding of the $(T, Z_j, M_j) - Y$ relationship: $Y(t, z_j, m_j) \perp\!\!\!\perp T \mid \boldsymbol{X}=\boldsymbol{x}$ for all levels of t, z_j, m_j, and $\boldsymbol{x}$, $Y(t, z_j, m_j) \perp\!\!\!\perp Z_j \mid T=t, \boldsymbol{X}=\boldsymbol{x}$ for all levels of t, z_j, m_j, and $Y(t, z_j, m_j) \perp\!\!\!\perp M_j \mid T=t, Z_j=z_j, \boldsymbol{X}=\boldsymbol{x}$ for all levels of t, z_j, m_j;
B2: No unmeasured confounding of the $T - Z_j$ or $(T, Z_j) - M_j$ relationships: $Z_j(t) \perp\!\!\!\perp T \mid \boldsymbol{X}=\boldsymbol{x}$ for all levels of t, $M_j(t, z_j) \perp\!\!\!\perp Z_j \mid T=t, \boldsymbol{X}=\boldsymbol{x}$ for all levels of t, z_j, and $M_j(t, z_j) \perp\!\!\!\perp T \mid \boldsymbol{X}=\boldsymbol{x}$ for all levels of t, z_j.

Using the notation in the main text, we can write $E\{Y(1, Z_j(1), M_j(1, Z_j(1)))|\boldsymbol{X}=\boldsymbol{x}\}$ as

$$
\begin{aligned}
&E\{Y(1, Z_j(1), M_j(1, Z_j(1)))|\boldsymbol{X}=\boldsymbol{x}\} \\
&= \sum_{m_j} E\{Y(1,1,m_j) \mid Z_j(1)=1, M_j(1, Z_j(1))) = m_j, \boldsymbol{x}\} p\{M_j(1, Z_j(1)) = m_j \mid Z_j(1)=1, \boldsymbol{x}\} p\{Z_j(1)=1|\boldsymbol{x}\} \\
&+ \sum_{m_j} E\{Y(1,0,m_j) \mid Z_j(1)=0, M_j(1, Z_j(1))) = m_j, \boldsymbol{x}\} p\{M_j(1, Z_j(1)) = m_j \mid Z_j(1)=0, \boldsymbol{x}\} p\{Z_j(1)=0|\boldsymbol{x}\}. \quad (1)
\end{aligned}
$$

Given above assumptions, it is straightforward to demonstrate the identifiability of the first term in equation (1), as illustrated

$$
\begin{aligned}
&E\{Y(1,1,m_j) \mid Z_j(1)=1, M_j(1,Z_j(1)))=m_j, \boldsymbol{X}=\boldsymbol{x}\} \\
&\overset{\textbf{B1}}{=} E\{Y(1,1,m_j) \mid T=1, Z_j(1)=1, M_j(1,Z_j(1))=m_j, \boldsymbol{X}=\boldsymbol{x}\} \\
&= E\{Y \mid T=1, Z_j=1, M_j=m_j, \boldsymbol{X}=\boldsymbol{x}\}.
\end{aligned}
$$

Similarly, we can derive $E\{Y(1,0,m_j) \mid Z_j(1)=0, M_j(1,Z_j(1)))=m_j, \boldsymbol{X}=\boldsymbol{x}\}$, where

$$
\begin{aligned}
&E\{Y(1,0,m_j) \mid Z_j(1)=0, M_j(1,Z_j(1)))=m_j, \boldsymbol{X}=\boldsymbol{x}\} \\
&\overset{\textbf{B1}}{=} E\{Y(1,0,m_j) \mid T=1, Z_j(1)=0, M_j(1,Z_j(1))=m_j, \boldsymbol{X}=\boldsymbol{x}\} \\
&= E\{Y \mid T=1, Z_j=0, M_j=m_j, \boldsymbol{X}=\boldsymbol{x}\}.
\end{aligned}
$$

For the second term of equation (1), we can easily derive $p\{M_j(1,Z_j(1))=m_j \mid Z_j(1)=1,\boldsymbol{x}\}$ and $p\{M_j(1,Z_j(1))=m_j \mid Z_j(1)=0,\boldsymbol{x}\}$, where

$$
\begin{aligned}
&p\{M_j(1,Z_j(1))=m_j \mid Z_j(1)=1, \boldsymbol{X}=\boldsymbol{x}\} \\
&= p\{M_j(1,1)=m_j \mid Z_j(1)=1, \boldsymbol{X}=\boldsymbol{x}\} \\
&\overset{\textbf{B2}}{=} p\{M_j(1,1)=m_j \mid T=1, Z_j(1)=1, \boldsymbol{X}=\boldsymbol{x}\} \\
&= p\{M_j=m_j \mid T=1, Z_j=1, \boldsymbol{X}=\boldsymbol{x}\},
\end{aligned}
$$

and

$$
\begin{aligned}
&p\{M_j(1,Z_j(1))=m_j \mid Z_j(1)=0, \boldsymbol{X}=\boldsymbol{x}\} \\
&= p\{M_j(1,0)=m_j \mid Z_j(1)=0, \boldsymbol{X}=\boldsymbol{x}\} \\
&\overset{\textbf{B2}}{=} p\{M_j(1,0)=m_j \mid T=1, Z_j(1)=0, \boldsymbol{X}=\boldsymbol{x}\} \\
&= p\{M_j=m_j \mid T=1, Z_j=0, \boldsymbol{X}=\boldsymbol{x}\}.
\end{aligned}
$$

Therefore,

$$
\begin{aligned}
&E\{Y(1,Z_j(1),M_j(1,Z_j(1)))|\boldsymbol{X}=\boldsymbol{x}\} \\
&= \sum_{m_j} E\{Y \mid T=1, Z_j=1, M_j=m_j, \boldsymbol{X}=\boldsymbol{x}\} P(M_j=m_j \mid T=1, Z_j=1, \boldsymbol{X}=\boldsymbol{x}) P(Z_j=1 \mid T=1, \boldsymbol{X}=\boldsymbol{x}) \\
&\quad + \sum_{m_j} E\{Y \mid T=1, Z_j=0, M_j=m_j, \boldsymbol{X}=\boldsymbol{x}\} P(M_j=m_j \mid T=1, Z_j=0, \boldsymbol{X}=\boldsymbol{x}) P(Z_j=0 \mid T=1, \boldsymbol{X}=\boldsymbol{x}) \\
&= \left\{\beta_0+\beta_1+\boldsymbol{\beta}_x^\top \boldsymbol{x}+\beta_{mj} E(M_j \mid T=1, Z_j=1, \boldsymbol{X}=\boldsymbol{x})\right\} P(Z_j=1 \mid T=1, \boldsymbol{X}=\boldsymbol{x}) \\
&\quad + \left\{\beta_0+\beta_1+\boldsymbol{\beta}_x^\top \boldsymbol{x}++\beta_{mj} E(M_j \mid T=1, Z_j=0, \boldsymbol{X}=\boldsymbol{x})\right\} P(Z_j=0 \mid T=1, \boldsymbol{X}=\boldsymbol{x}) \\
&= \beta_0+\beta_1+\boldsymbol{\beta}_x^\top \boldsymbol{x}+\beta_{mj}\exp(\alpha_{0j}+\alpha_{1j}+\boldsymbol{\alpha}_{xj}^\top \boldsymbol{x}) P(Z_j=0 \mid T=1, \boldsymbol{X}=\boldsymbol{x}).
\end{aligned}
$$

Similarly, we can easily derive $E\{Y(1,Z_j(0),M_j(1,Z_j(0)))|\boldsymbol{X}=\boldsymbol{x}\}$ and $E\{Y(1,Z_j(0),M_j(0,Z_j(0)))|\boldsymbol{X}=\boldsymbol{x}\}$, where

$$
\begin{aligned}
&E\{Y(1,Z_j(0),M_j(1,Z_j(0)))|\boldsymbol{X}=\boldsymbol{x}\} \\
&= \sum_{m_j} E\{Y \mid T=1, Z_j=1, M_j=m_j, \boldsymbol{X}=\boldsymbol{x}\} P(M_j=m_j \mid T=1, Z_j=1, \boldsymbol{X}=\boldsymbol{x}) P(Z_j=1 \mid T=0, \boldsymbol{X}=\boldsymbol{x}) \\
&\quad + \sum_{m_j} E\{Y \mid T=1, Z_j=0, M_j=m_j, \boldsymbol{X}=\boldsymbol{x}\} P(M_j=m_j \mid T=1, Z_j=0, \boldsymbol{X}=\boldsymbol{x}) P(Z_j=0 \mid T=0, \boldsymbol{X}=\boldsymbol{x}) \\
&= \left\{\beta_0+\beta_1+\boldsymbol{\beta}_x^\top \boldsymbol{x}+\beta_{mj} E(M_j \mid T=1, Z_j=1, \boldsymbol{X}=\boldsymbol{x})\right\} P(Z_j=1 \mid T=0, \boldsymbol{X}=\boldsymbol{x}) \\
&\quad + \left\{\beta_0+\beta_1+\boldsymbol{\beta}_x^\top \boldsymbol{x}+\beta_{mj} E(M_j \mid T=1, Z_j=0, \boldsymbol{X}=\boldsymbol{x})\right\} P(Z_j=0 \mid T=0, \boldsymbol{X}=\boldsymbol{x}) \\
&= \beta_0+\beta_1+\boldsymbol{\beta}_x^\top \boldsymbol{x}+\beta_{mj}\exp(\alpha_{0j}+\alpha_{1j}+\boldsymbol{\alpha}_{xj}^\top \boldsymbol{x}) P(Z_j=0 \mid T=0, \boldsymbol{X}=\boldsymbol{x}),
\end{aligned}
$$

and

$$
\begin{aligned}
&E\{Y(1, Z_j(0), M_j(0, Z_j(0)))|\boldsymbol{X}=\boldsymbol{x}\} \\
&=\sum_{m_j} E\{Y \mid T=1, Z_j=1, M_j=m_j, \boldsymbol{X}=\boldsymbol{x}\} P(M_j=m_j \mid T=0, Z_j=1, \boldsymbol{X}=\boldsymbol{x}) P(Z_j=1 \mid T=0, \boldsymbol{X}=\boldsymbol{x}) \\
&\quad+\sum_{m_j} E\{Y \mid T=1, Z_j=0, M_j=m_j, \boldsymbol{X}=\boldsymbol{x}\} P(M_j=m_j \mid T=0, Z_j=0, \boldsymbol{X}=\boldsymbol{x}) P(Z_j=0 \mid T=0, \boldsymbol{X}=\boldsymbol{x}) \\
&=\left\{\beta_0+\beta_1+\boldsymbol{\beta}_x^{\top} \boldsymbol{x}+\beta_{mj} E(M_j \mid T=0, Z_j=1, \boldsymbol{X}=\boldsymbol{x})\right\} P(Z_j=1 \mid T=0, \boldsymbol{X}=\boldsymbol{x}) \\
&\quad+\left\{\beta_0+\beta_1+\boldsymbol{\beta}_x^{\top} \boldsymbol{x}+\beta_{mj} E(M_j \mid T=0, Z_j=0, \boldsymbol{X}=\boldsymbol{x})\right\} P(Z_j=0 \mid T=0, \boldsymbol{X}=\boldsymbol{x}) \\
&=\beta_0+\beta_1+\boldsymbol{\beta}_x^{\top} \boldsymbol{x}+\beta_{mj} \exp(\alpha_{0j}+\boldsymbol{\alpha}_{xj}^{\top} \boldsymbol{x}) P(Z_j=0 \mid T=0, \boldsymbol{X}=\boldsymbol{x}),
\end{aligned}
$$

Based on the above equations, we can derive the indirect effect NIEP_j and NIEA_j, respectively, where

$$
\begin{aligned}
\mathrm{NIEP}_j &= E\{Y(1, Z_j(1), M_j(1, Z_j(1)))|\boldsymbol{X}=\boldsymbol{x}\} - E\{Y(1, Z_j(0), M_j(1, Z_j(0)))|\boldsymbol{X}=\boldsymbol{x}\} \\
&= \left\{\beta_0+\beta_1+\boldsymbol{\beta}_x^{\top} \boldsymbol{x}+\beta_{mj} \exp(\alpha_{0j}+\alpha_{1j}+\boldsymbol{\alpha}_{xj}^{\top} \boldsymbol{x}) P(Z_j=0 \mid T=1, \boldsymbol{X}=\boldsymbol{x})\right\} \\
&\quad -\left\{\beta_0+\beta_1+\boldsymbol{\beta}_x^{\top} \boldsymbol{x}+\beta_{mj} \exp(\alpha_{0j}+\alpha_{1j}+\boldsymbol{\alpha}_{xj}^{\top} \boldsymbol{x}) P(Z_j=0 \mid T=0, \boldsymbol{X}=\boldsymbol{x})\right\} \\
&= \beta_{mj} \exp(\alpha_{0j}+\alpha_{1j}+\boldsymbol{\alpha}_{xj}^{\top} \boldsymbol{x}) P(Z_j=0 \mid T=1, \boldsymbol{X}=\boldsymbol{x}) - \beta_{mj} \exp(\alpha_{0j}+\alpha_{1j}+\boldsymbol{\alpha}_{xj}^{\top} \boldsymbol{x}) P(Z_j=0 \mid T=0, \boldsymbol{X}=\boldsymbol{x}) \\
&= \beta_{mj} \exp(\alpha_{0j}+\alpha_{1j}+\boldsymbol{\alpha}_{xj}^{\top} \boldsymbol{x}) \left\{P(Z_j=0 \mid T=1, \boldsymbol{X}=\boldsymbol{x}) - P(Z_j=0 \mid T=0, \boldsymbol{X}=\boldsymbol{x})\right\} \\
&= \beta_{mj} \left\{\frac{\exp(\alpha_{0j}+\alpha_{1j}+\boldsymbol{\alpha}_{xj}^{\top} \boldsymbol{x})}{1+\exp(\gamma_{0j}+\gamma_{1j}+\boldsymbol{\gamma}_{xj}^{\top} \boldsymbol{x})} - \frac{\exp(\alpha_{0j}+\alpha_{1j}+\boldsymbol{\alpha}_{xj}^{\top} \boldsymbol{x})}{1+\exp(\gamma_{0j}+\boldsymbol{\gamma}_{xj}^{\top} \boldsymbol{x})}\right\},
\end{aligned}
$$

and

$$
\begin{aligned}
\mathrm{NIEA}_j &= E\{Y(1, Z_j(0), M_j(1, Z_j(0)))|\boldsymbol{X}=\boldsymbol{x}\} - E\{Y(1, Z_j(0), M_j(0, Z_j(0)))|\boldsymbol{X}=\boldsymbol{x}\} \\
&= \left\{\beta_0+\beta_1+\boldsymbol{\beta}_x^{\top} \boldsymbol{x}+\beta_{mj} \exp(\alpha_{0j}+\alpha_{1j}+\boldsymbol{\alpha}_{xj}^{\top} \boldsymbol{x}) P(Z_j=0 \mid T=0, \boldsymbol{X}=\boldsymbol{x})\right\} \\
&\quad -\left\{\beta_0+\beta_1+\boldsymbol{\beta}_x^{\top} \boldsymbol{x}+\beta_{mj} \exp(\alpha_{0j}+\boldsymbol{\alpha}_{xj}^{\top} \boldsymbol{x}) P(Z_j=0 \mid T=0, \boldsymbol{X}=\boldsymbol{x})\right\} \\
&= \beta_{mj} P(Z_j=0 \mid T=0, \boldsymbol{X}=\boldsymbol{x}) \left\{\exp(\alpha_{0j}+\alpha_{1j}+\boldsymbol{\alpha}_{xj}^{\top} \boldsymbol{x}) - \exp(\alpha_{0j}+\boldsymbol{\alpha}_{xj}^{\top} \boldsymbol{x})\right\} \\
&= \beta_{mj} \left\{\frac{\exp(\alpha_{0j}+\alpha_{1j}+\boldsymbol{\alpha}_{xj}^{\top} \boldsymbol{x})}{1+\exp(\gamma_{0j}+\boldsymbol{\gamma}_{xj}^{\top} \boldsymbol{x})} - \frac{\exp(\alpha_{0j}+\boldsymbol{\alpha}_{xj}^{\top} \boldsymbol{x})}{1+\exp(\gamma_{0j}+\boldsymbol{\gamma}_{xj}^{\top} \boldsymbol{x})}\right\}.
\end{aligned}
$$

With NIEP_j and NIEA_j defined, we have the decomposition: $\mathrm{NIE}_j = \mathrm{NIEP}_j + \mathrm{NIEA}_j$, so that the overall natural indirect effect decomposes into the sum of the effect through Z_j and not through Z_j.

II. Additional simulation studies

As described in the main text, to evaluate the robustness of the proposed CRAmed method against violations of the ZINB model assumption, microbiome data $\mathbf{M} = (M_{ij})$ were generated from the hurdle Poisson and hurdle NB models. The treatment T_i was sampled from a Bernoulli distribution with parameter 0.5. Given the treatment T_i and the mediators $\boldsymbol{M}_i$, we generated the outcome Y_i from a normal distribution. Additionally, to explore the effects of sample size (n) and the dimension of mediators (m) on performance, we examined four different combinations with $n \in \{100, 200\}$ and $m \in \{100, 1000\}$. The results, presented in Figures S1–S4, demonstrate that despite model misspecification, the proposed CRAmed method consistently outperforms others in identifying causal taxa, highlighting its robustness.

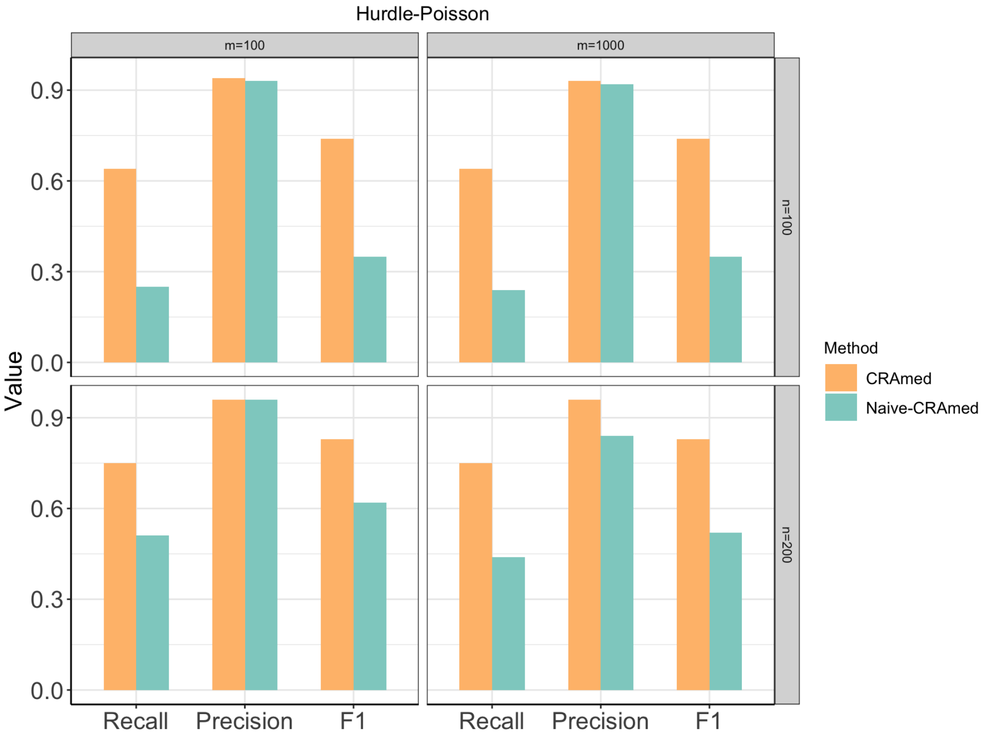

Figure S1: Comparison of Recall, Precision, and F1 score for the Naive-CRAmed and CRAmed methods using microbiome data generated from the hurdle Poisson model. Sample size $n \in \{100, 200\}$ and number of taxa $m \in \{100, 1000\}$.

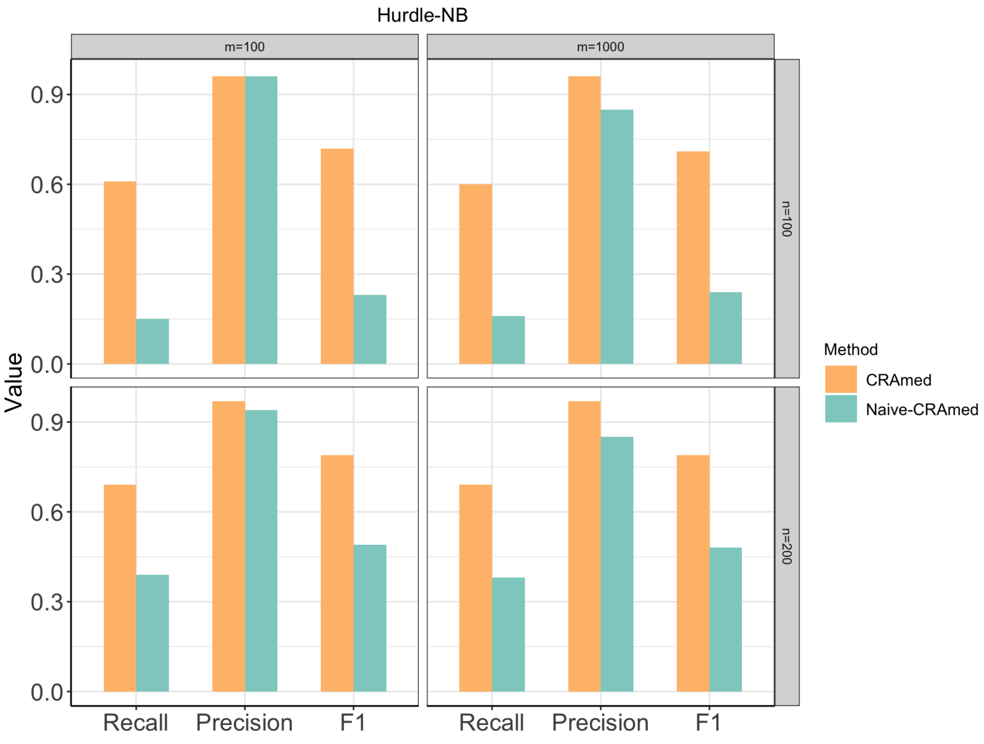

Figure S2: Comparison of Recall, Precision, and F1 score for the Naive-CRAmed and CRAmed methods using microbiome data generated from the hurdle NB model. Sample size $n \in \{100, 200\}$ and number of taxa $m \in \{100, 1000\}$.

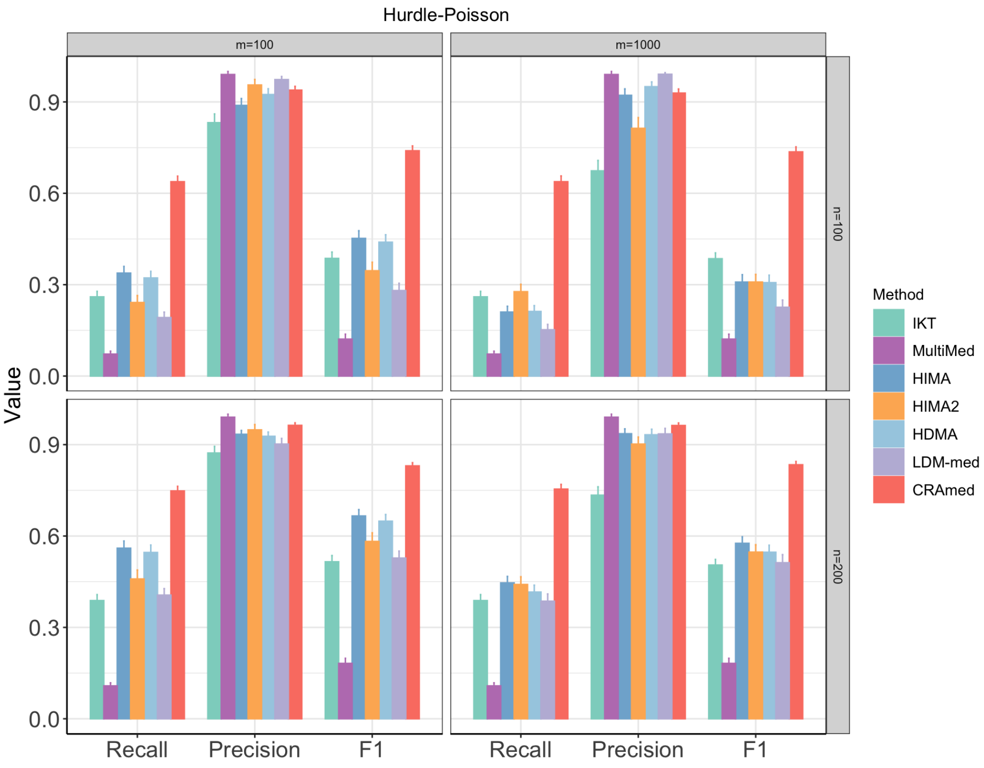

Figure S3: Comparison of Recall, Precision, and F1 score for different mediation analysis methods using microbiome data generated from the hurdle Poisson model. Sample size $n \in \{100, 200\}$ and number of taxa $m \in \{100, 1000\}$.

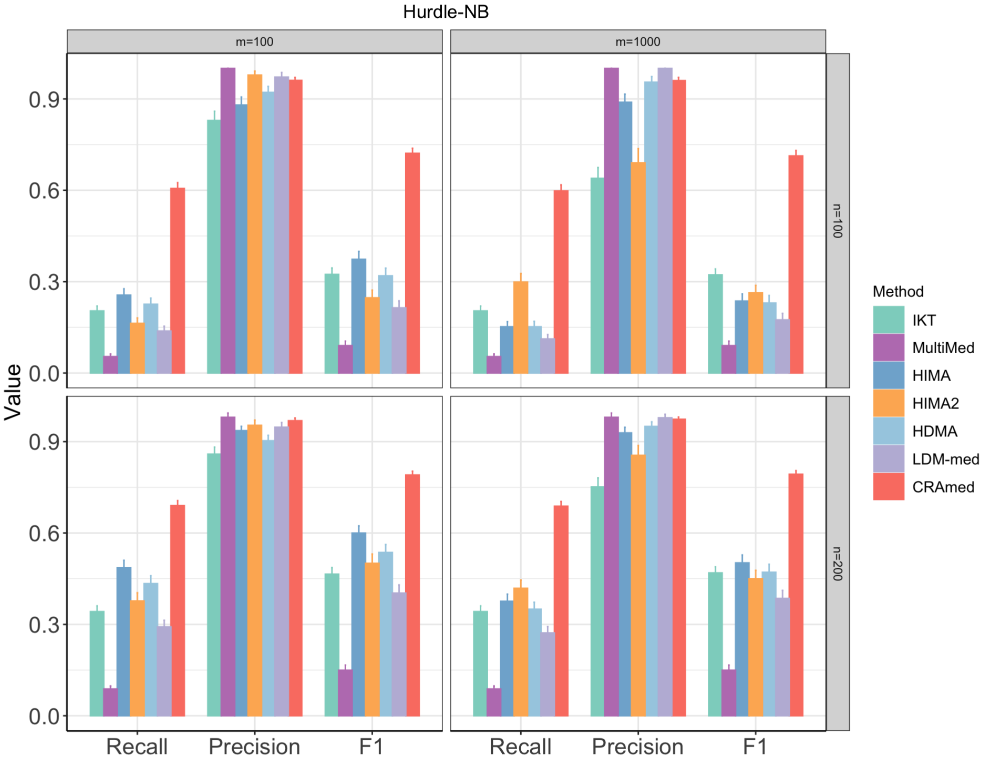

Figure S4: Comparison of Recall, Precision, and F1 score for different mediation analysis methods using microbiome data generated from the hurdle NB model. Sample size $n \in \{100, 200\}$ and number of taxa $m \in \{100, 1000\}$.

To comprehensively evaluate the robustness and reliability of CRAmed and its competitors, we have expanded the simulation study to include a broader range of settings for generating unmeasured confounders, systematically violating each of the A1–A4 assumptions to varying degrees. To violate the A1 assumption, we generated the treatment T_i from a Bernoulli distribution with parameter η_{ij}, where

$$\text{logit}(\eta_{ij}) \quad = \quad \boldsymbol{\Delta}_{uj}^{\top}\boldsymbol{X}_i^u.$$

Here, $\boldsymbol{X}_i^u$ denotes a d-vector of unobserved confounders. The microbiome data $\mathbf{M} = (M_{ij})$ were generated from the ZINB model. Given the treatment T_i and the mediators $\boldsymbol{M}_i$, we generated the outcome Y_i from a normal distribution $N(\mu_i, \sigma^2)$, where

$$\mu_i = \beta_0 + \beta_1 T_i + \boldsymbol{\beta}_m^{\top}\boldsymbol{M}_i + \boldsymbol{\beta}_u^{\top}\boldsymbol{X}_i^u.$$

With $d = 3$, the unobserved confounders were sampled independently from $N(0, 1)$. To evaluate how these confounders affect the performance of various methods, we sampled $\boldsymbol{\Delta}_{uj}$ and $\boldsymbol{\beta}_u$ independently from $N(0.2h, 0.5)$, where $h \in \{1, 3, 9\}$. The remaining settings were consistent with the previous examples, except that the regression coefficients of $\boldsymbol{\gamma}_1$, $\boldsymbol{\alpha}_1$, and those of $\boldsymbol{\beta}_m$, were drawn from $N(-5, 1)$ and $N(5, 1)$, respectively.

The results are shown in Figures S5-S7. When unmeasured confounders influenced the relationship between treatment and outcome, we observe that the performance of all methods deteriorated as the extent of violation (h) increased. Nevertheless, CRAmed consistently achieves the highest F1 score while maintaining competitively high precision across all settings, highlighting the robustness of CRAmed in the presence of unobserved confounders between treatment and outcome.

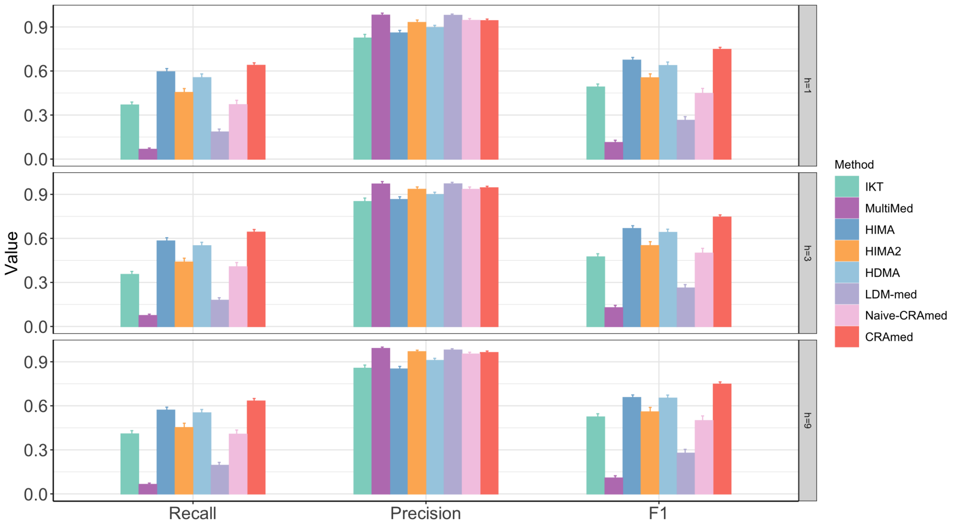

Figure S5: Comparison of Recall, Precision, and F1 score in the sensitivity analysis using microbiome data generated from the ZINB model, with unobserved confounders present in the relationship between treatment and outcome. Sample size $n = 100$ and number of taxa $m = 100$.

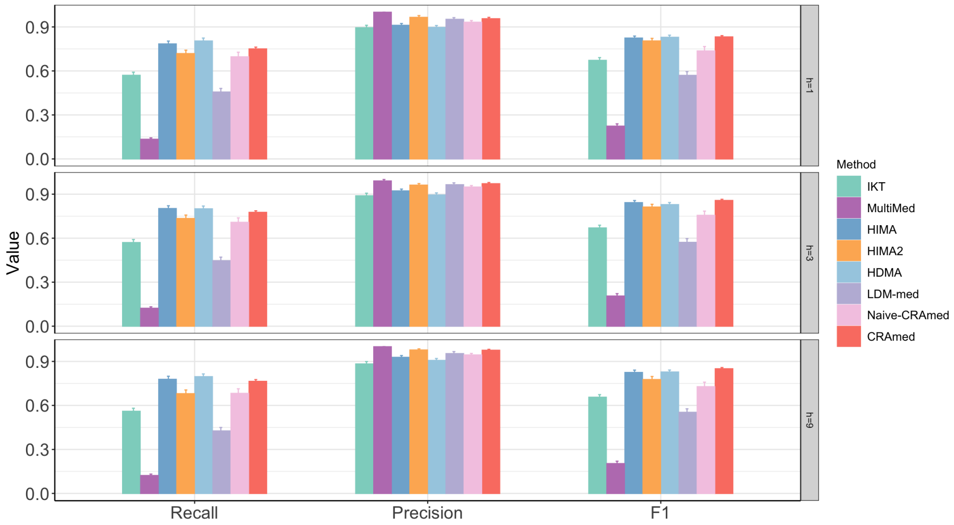

Figure S6: Comparison of Recall, Precision, and F1 score in the sensitivity analysis using microbiome data generated from the ZINB model, with unobserved confounders present in the relationship between treatment and outcome. Sample size $n = 200$ and number of taxa $m = 100$.

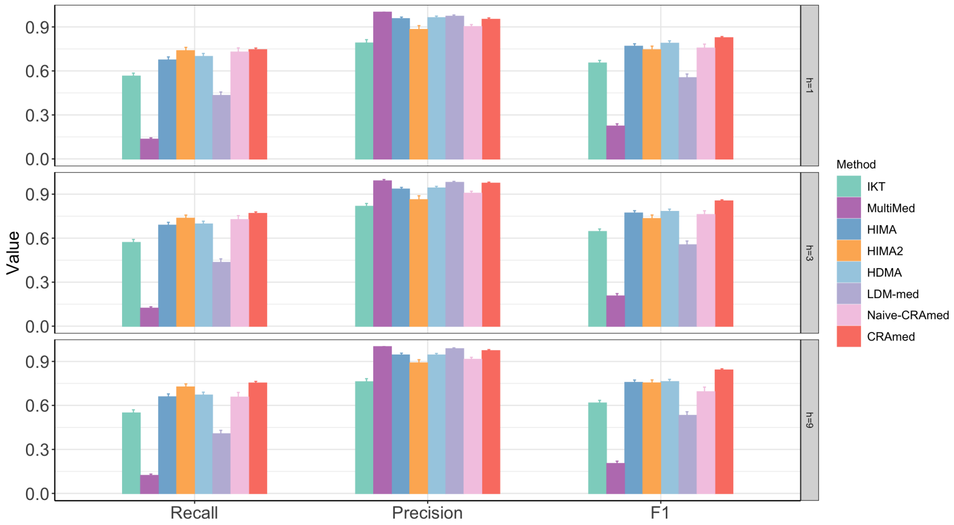

Figure S7: Comparison of Recall, Precision, and F1 score in the sensitivity analysis using microbiome data generated from the ZINB model, with unobserved confounders present in the relationship between treatment and outcome. Sample size $n = 200$ and number of taxa $m = 1000$.

To violate the A2 assumption, we sampled the treatment T_i from a Bernoulli distribution with parameter 0.5 and generated microbiome data $\mathbf{M} = (M_{ij}$ from the ZINB model, with the link functions specified as

$$\begin{aligned} \text{logit}(\pi_{ij}) &= \gamma_{0j} + \gamma_{1j} T_i + \boldsymbol{\gamma}_{uj}^\top \boldsymbol{X}_i^u, \\ \log(\lambda_{ij}) &= \log(S_i) + \alpha_{0j} + \alpha_{1j} T_i + \boldsymbol{\alpha}_{uj}^\top \boldsymbol{X}_i^u, \end{aligned}$$

for $i = 1, \ldots, n$, $j = 1, \ldots, m$, where $\boldsymbol{X}_i^u$ denotes a d-vector of unobserved confounders. Given the microbiome data $\boldsymbol{M}_i$ and treatment T_i, we generated the outcome Y_i from a normal distribution $N(\mu_i, \sigma^2)$, where

$$\mu_i = \beta_0 + \beta_1 T_i + \boldsymbol{\beta}_m^\top \boldsymbol{M}_i + \boldsymbol{\beta}_u^\top \boldsymbol{X}_i^u.$$

With $d = 3$, the unobserved confounders were sampled independently from $N(0, 1)$. We sampled $\boldsymbol{\gamma}_{uj}$, $\boldsymbol{\alpha}_{uj}$, and $\boldsymbol{\beta}_u$ independently from $N(0.2h, 0.5)$, where $h \in \{1, 3, 9\}$. The remaining scenarios were consistent with the earlier examples. The simulation results are presented in Figures S8–S11. The performance of all methods deteriorated as the extent of violation (h) increased. Nevertheless, CRAmed outperformed its competitors in terms of Recall and F1 score, except for $h = 9$, in which case all methods performed poorly. In summary, CRAmed remained competitive in the presence of unobserved confounders between the mediator and outcome.

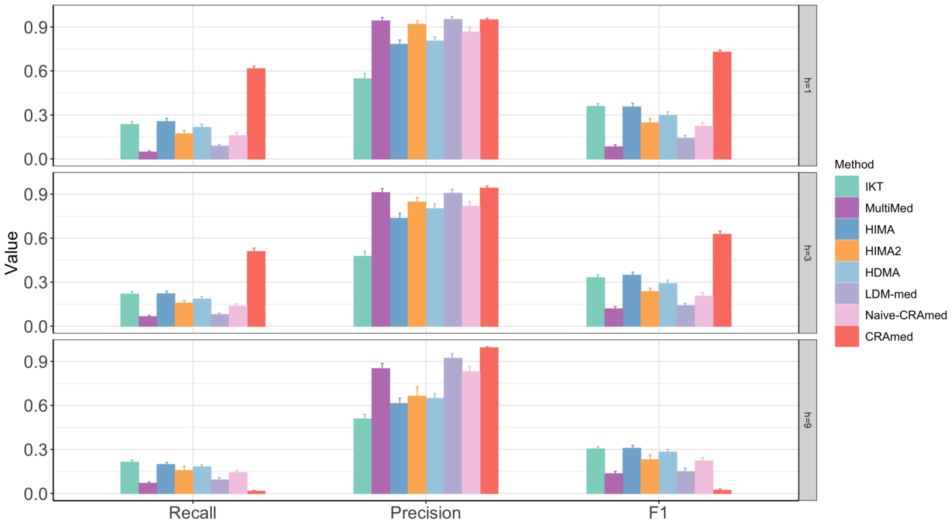

Figure S8: Comparison of Recall, Precision, and F1 score in the sensitivity analysis using microbiome data generated from the ZINB model, with unobserved confounders present in the relationship between mediator and outcome. Sample size $n = 100$ and number of taxa $m = 100$.

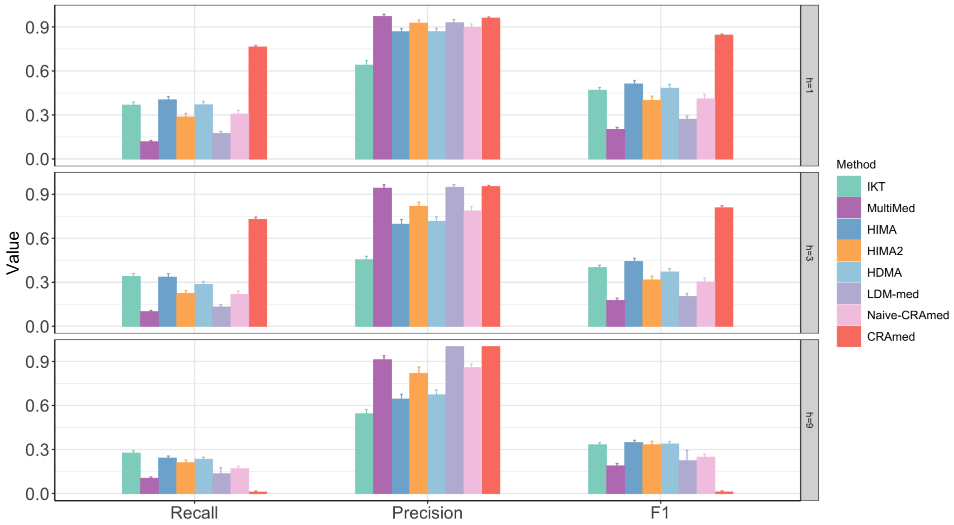

Figure S9: Comparison of Recall, Precision, and F1 score in the sensitivity analysis using microbiome data generated from the ZINB model, with unobserved confounders present in the relationship between mediator and outcome. Sample size $n = 200$ and number of taxa $m = 100$.

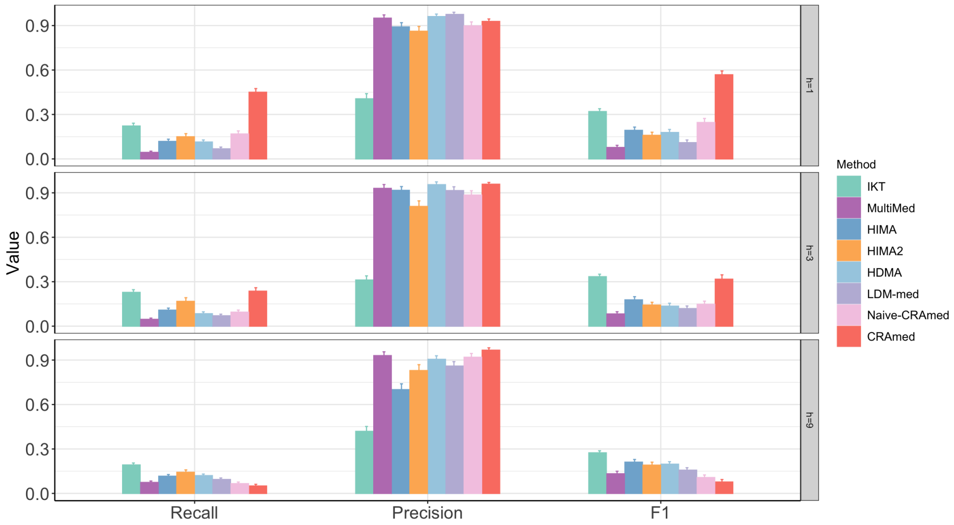

Figure S10: Comparison of Recall, Precision, and F1 score in the sensitivity analysis using microbiome data generated from the ZINB model, with unobserved confounders present in the relationship between mediator and outcome. Sample size $n = 100$ and number of taxa $m = 1000$.

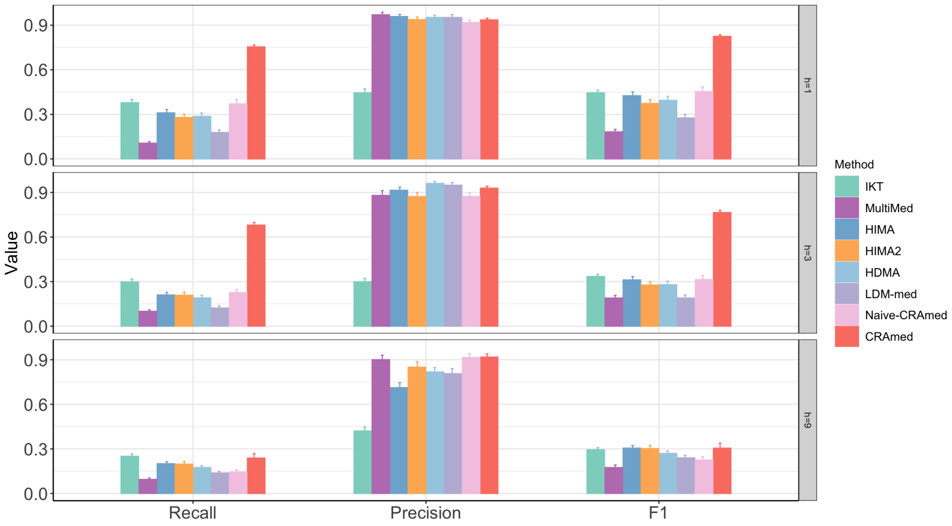

Figure S11: Comparison of Recall, Precision, and F1 score in the sensitivity analysis using microbiome data generated from the ZINB model, with unobserved confounders present in the relationship between mediator and outcome. Sample size $n = 200$ and number of taxa $m = 1000$.

To violate the A3 assumption, we first generated the treatment T_i from a Bernoulli distribution with parameter η_{ij}, where

$$\text{logit}(\eta_{ij}) = \boldsymbol{\Delta}_{uj}^\top \boldsymbol{X}_i^u.$$

And then we generated microbiome data $\boldsymbol{M}_i$ from the ZINB model, with the link functions specified as

$$\begin{aligned}\text{logit}(\pi_{ij}) &= \gamma_{0j} + \gamma_{1j} T_i + \boldsymbol{\gamma}_{uj}^\top \boldsymbol{X}_i^u,\\ \log(\lambda_{ij}) &= \log(S_i) + \alpha_{0j} + \alpha_{1j} T_i + \boldsymbol{\alpha}_{uj}^\top \boldsymbol{X}_i^u.\end{aligned}$$

Given the microbiome data $\boldsymbol{M}_i$ and treatment T_i, we generated the outcome Y_i from a normal distribution $N(\mu_i, \sigma^2)$, where

$$\mu_i = \beta_0 + \beta_1 T_i + \boldsymbol{\beta}_m^\top \boldsymbol{M}_i.$$

We sampled $\boldsymbol{\Delta}_{uj}$, $\boldsymbol{\gamma}_{uj}$, and $\boldsymbol{\alpha}_{uj}$ independently from $N(0.2h, 0.5)$, where $h \in \{1, 3, 9\}$. The remaining scenarios were consistent with the earlier examples, and the simulation results are presented in Figures S12–S15. Again, as the extent of violation (h) increased, the performance of all methods declined. However, CRAmed consistently outperformed its competitors in terms of Recall and F1 score, except at $h = 9$, where all methods performed poorly. Overall, CRAmed demonstrates competitiveness, when unobserved confounders are present between treatment and mediator.

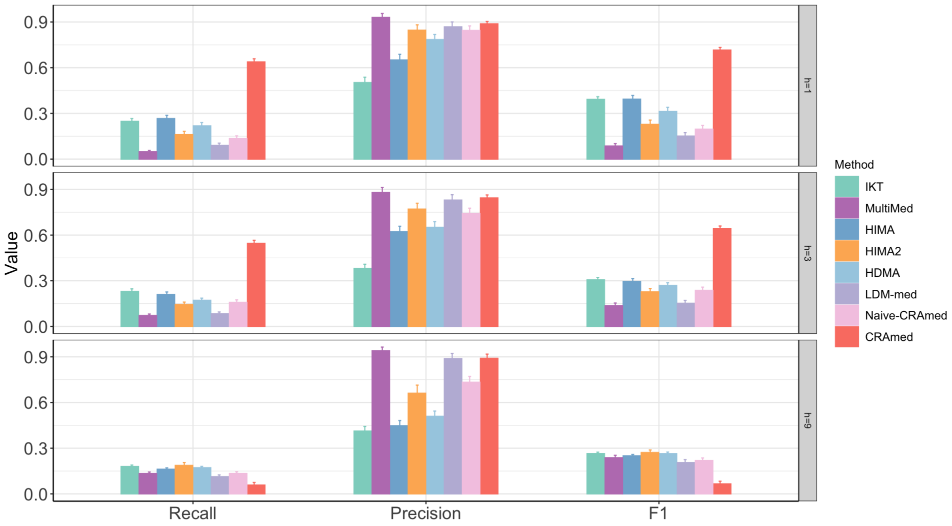

Figure S12: Comparison of Recall, Precision, and F1 score in the sensitivity analysis using microbiome data generated from the ZINB model, with unobserved confounders present in the relationship between treatment and mediator. Sample size $n = 100$ and number of taxa $m = 100$.

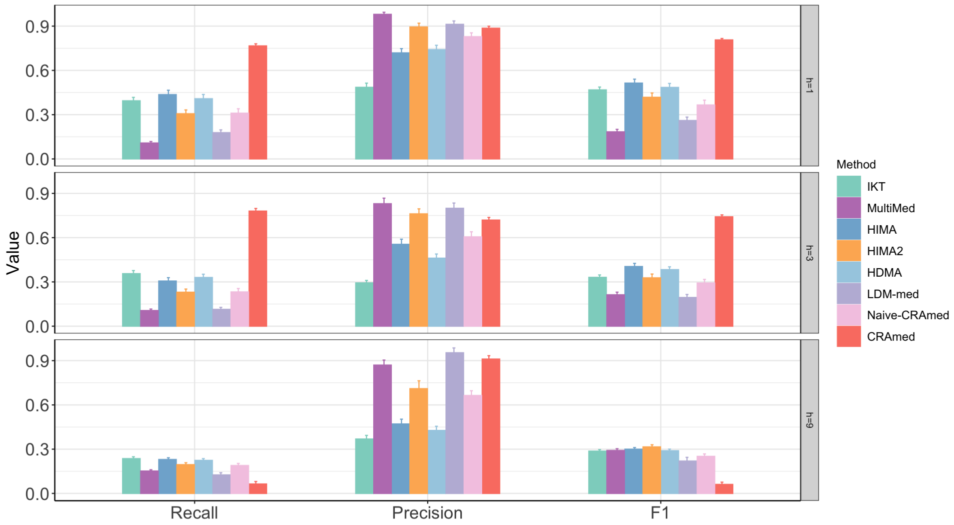

Figure S13: Comparison of Recall, Precision, and F1 score in the sensitivity analysis using microbiome data generated from the ZINB model, with unobserved confounders present in the relationship between treatment and mediator. Sample size $n = 200$ and number of taxa $m = 100$.

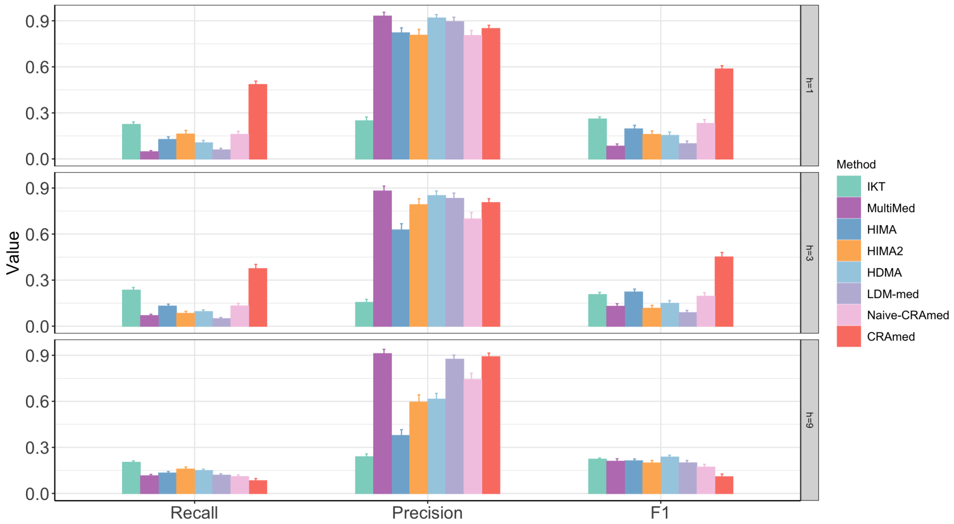

Figure S14: Comparison of Recall, Precision, and F1 score in the sensitivity analysis using microbiome data generated from the ZINB model, with unobserved confounders present in the relationship between treatment and mediator. Sample size $n = 100$ and number of taxa $m = 1000$.

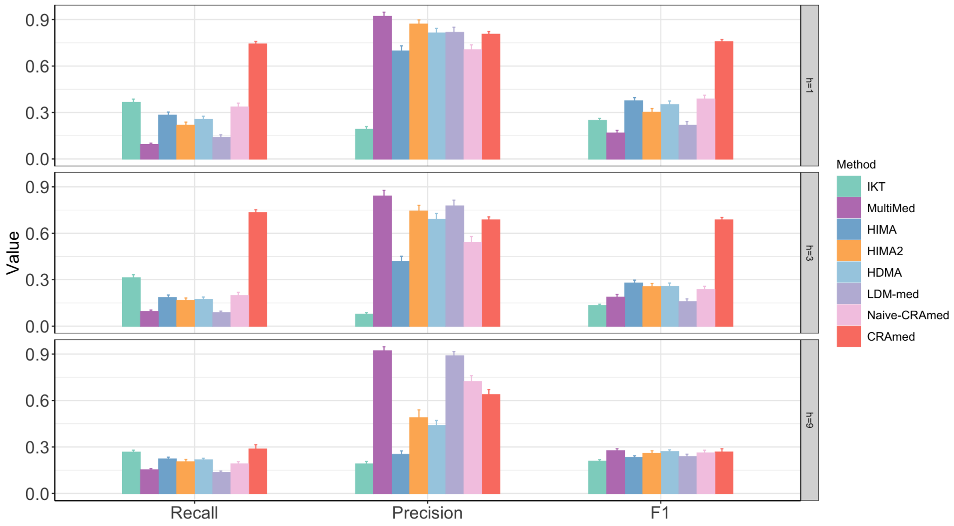

Figure S15: Comparison of Recall, Precision, and F1 score in the sensitivity analysis using microbiome data generated from the ZINB model, with unobserved confounders present in the relationship between treatment and mediator. Sample size $n = 200$ and number of taxa $m = 1000$.

To violate the A4 assumption, we first generated the treatment T_i from a Bernoulli distribution with parameter 0.5. The unobserved confounders $\boldsymbol{X}_i^u$ were then generated from a normal distribution $N(0.2T_i \times h, 1)$, where $h \in \{1, 3, 9\}$. Then we generated microbiome data $\boldsymbol{M}_i$ from the ZINB model, with the link functions specified as

$$\begin{aligned} \operatorname{logit}(\pi_{ij}) &= \gamma_{0j} + \gamma_{1j}T_i + \boldsymbol{\gamma}_{uj}^\top \boldsymbol{X}_i^u, \\ \log(\lambda_{ij}) &= \log(S_i) + \alpha_{0j} + \alpha_{1j}T_i + \boldsymbol{\alpha}_{uj}^\top \boldsymbol{X}_i^u. \end{aligned}$$

Given the microbiome data $\boldsymbol{M}_i$ and treatment T_i, we generated the outcome Y_i from a normal distribution $N(\mu_i, \sigma^2)$, where

$$\mu_i = \beta_0 + \beta_1 T_i + \boldsymbol{\beta}_m^\top \boldsymbol{M}_i + \boldsymbol{\beta}_u^\top \boldsymbol{X}_i^u.$$

Here, $\boldsymbol{\gamma}_{uj}$, $\boldsymbol{\alpha}_{uj}$, and $\boldsymbol{\beta}_u$ were sampled independently from $N(0.2h, 0.5)$, where $h \in \{1, 3, 9\}$. The remaining scenarios were consistent with the earlier examples, and the simulation results are presented in Figures S16–S19. CRAmed achieved the highest Recall and F1 score, except at $h = 9$, where all methods performed poorly. This further confirms CRAmed's robustness in the presence of unobserved confounders between the mediator and the outcome affected by the treatment.

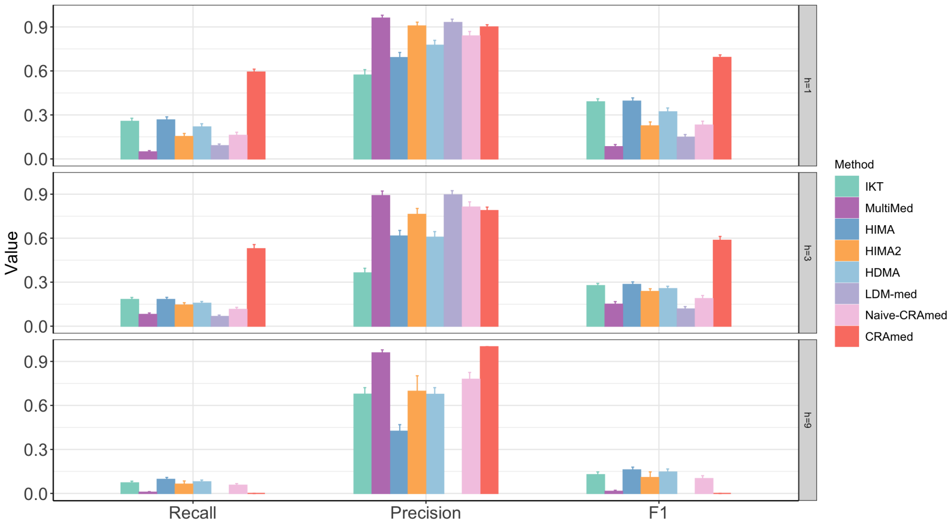

Figure S16: Comparison of Recall, Precision, and F1 score in the sensitivity analysis using microbiome data generated from the ZINB model, with unobserved confounders present in the relationship between mediator and outcome that is affected by the treatment. Sample size $n = 100$ and number of taxa $m = 100$.

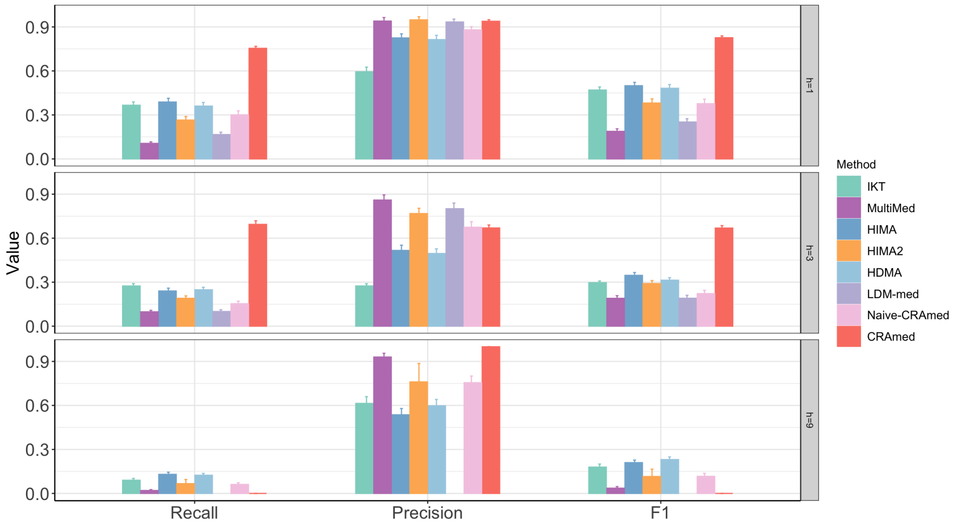

Figure S17: Comparison of Recall, Precision, and F1 score in the sensitivity analysis using microbiome data generated from the ZINB model, with unobserved confounders present in the relationship between mediator and outcome that is affected by the treatment. Sample size $n = 200$ and number of taxa $m = 100$.

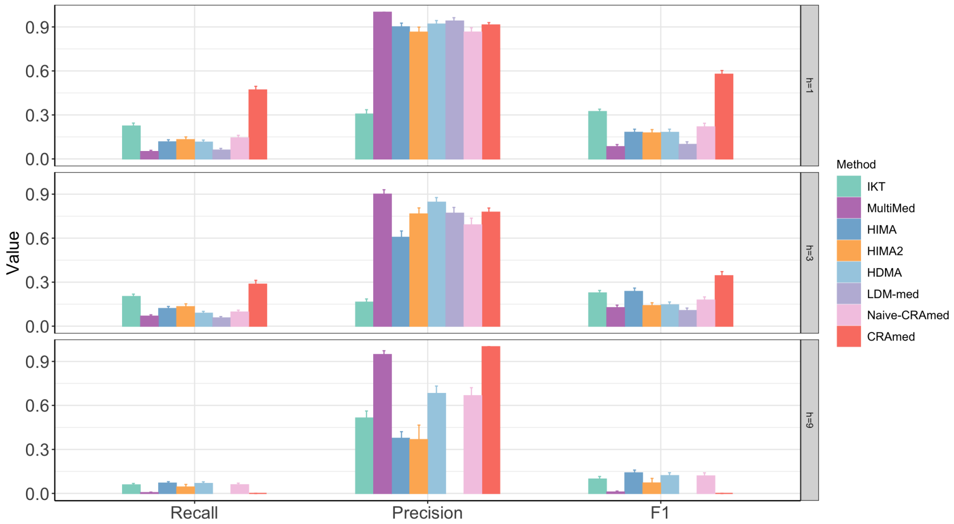

Figure S18: Comparison of Recall, Precision, and F1 score in the sensitivity analysis using microbiome data generated from the ZINB model, with unobserved confounders present in the relationship between mediator and outcome that is affected by the treatment. Sample size $n = 100$ and number of taxa $m = 1000$.

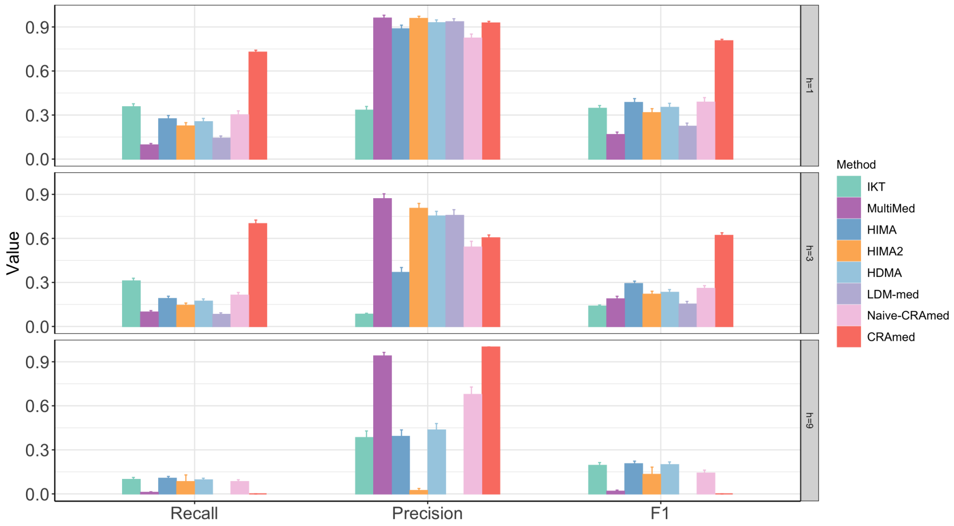

Figure S19: Comparison of Recall, Precision, and F1 score in the sensitivity analysis using microbiome data generated from the ZINB model, with unobserved confounders present in the relationship between mediator and outcome that is affected by the treatment. Sample size $n = 200$ and number of taxa $m = 1000$.

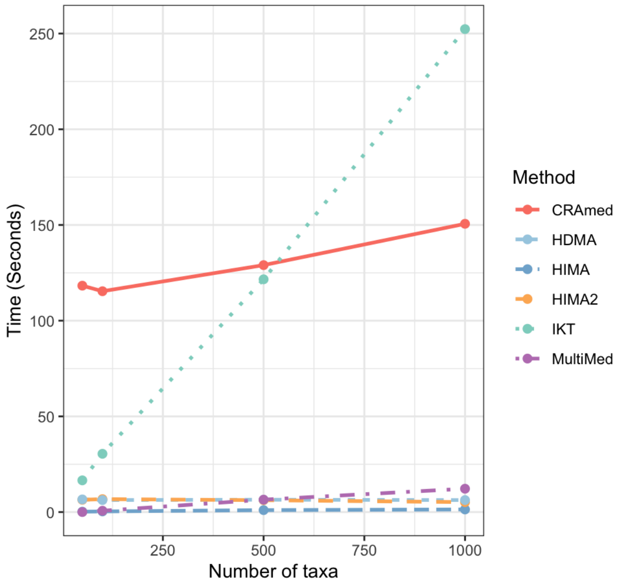

Figure S20: Time (seconds) versus the number of taxa averaged over 10 runs with data generated from the ZINB model ($n = 100$). Note that, due to the high computational cost, LDM-med is not included.

III. Preprocessing and additional results for the application studies in Section 3

A. Identification of microbial mediators of weight under different modes of delivery

We analyzed a publicly available gut microbiome dataset from a previous study [1], which includes clinical examinations and gut microbiome data from 1098 infants. Our investigation focused on the outcome variable of weight growth pace during the first year, aiming to explore whether the gut microbiome mediates the relationship between delivery mode and the infants' weight.

After filtering out taxa with a prevalence of less than 10%, we obtained a dataset with 876 taxa and 1098 samples. Figures S21 (a) and (b) depict distinct microbial profiles between the C-section and vaginal groups. Regarding alpha diversity, the vaginal group exhibits higher microbial richness and evenness, compared to the C-section group. In terms of Beta diversity, Bray–Curtis dissimilarity reveals that the C-section group has distinct community structures compared to the vaginal group. These community-level findings indicate that the microbiome may mediate the relationship between the mode of delivery and infants' weight.

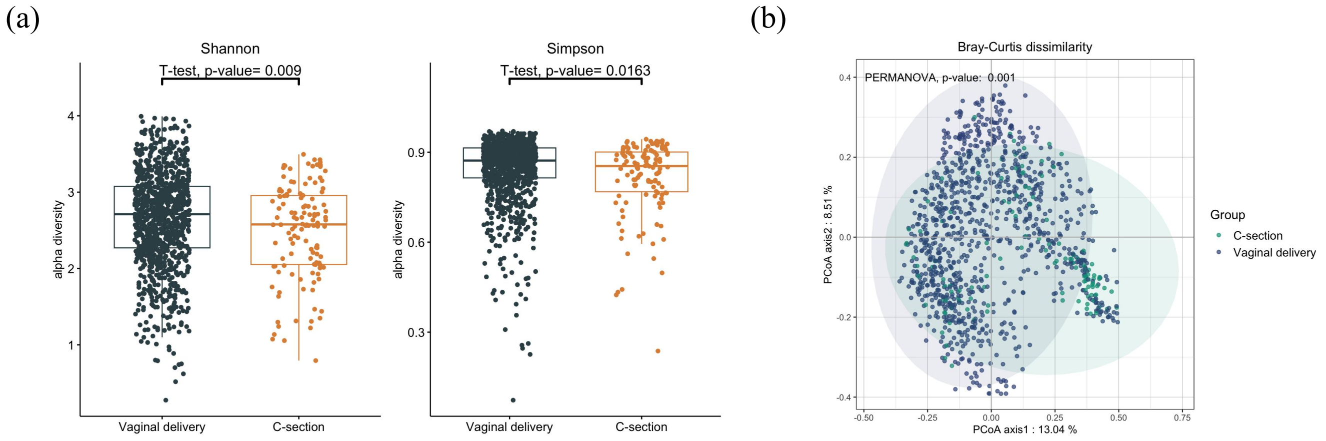

Figure S21: Alpha and beta diversity analyses for the DIABIUMMUNE dataset. (a) Box plots of alpha diversities (Shannon and Simpson indices) between the C-section and vaginal delivery groups. (b) PCoA plots using the Bray–Curtis dissimilarity.

B. Identification of microbial mediators of BMI and waist circumference under antibiotic treatment

We employed a dataset from the Guangdong Gut Microbiome Project (GGMP), a large community-based cross-sectional cohort conducted between 2015 and 2016, comprising 7009 participants with high-quality gut microbiome data [2]. Before proceeding, we filtered taxa with prevalence less than 10%. Due to the computational burden imposed by the large sample size, we randomly sampled an equal number of samples from non-antibiotics individuals as antibiotics individuals, resulting in 944 taxa across 894 samples.

Figure S22(a) demonstrates that the antibiotics and non-antibiotics groups exhibit distinct microbial profiles in terms of community diversity. From Figure S22(b), it is evident that the microbiome community is significantly correlated with BMI and waist circumference (WC). Hence, we posit that the microbiome may play a mediating role between antibiotics and phenotypes.

(a)

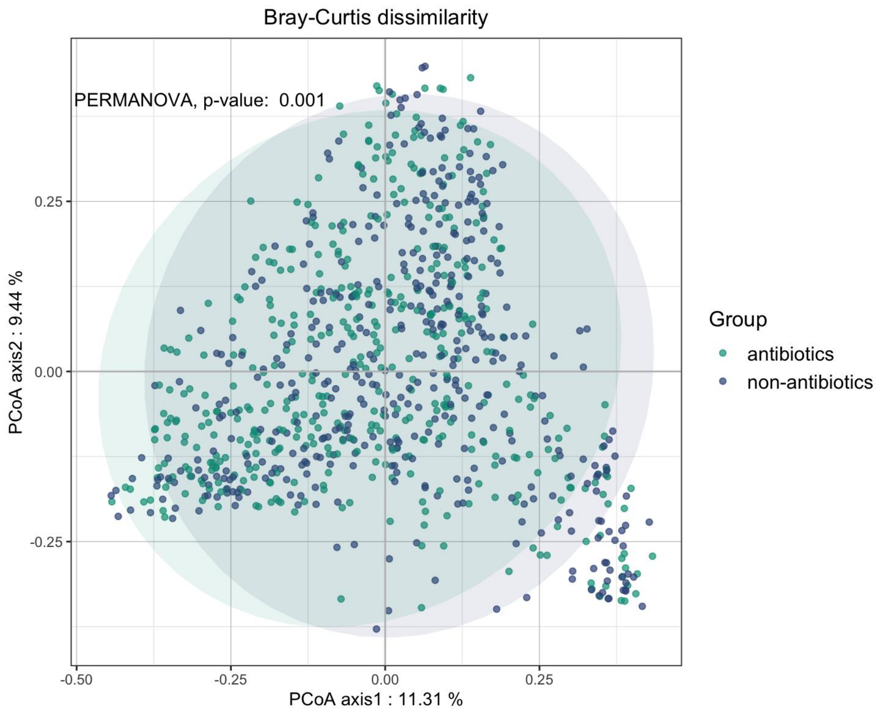

(b)

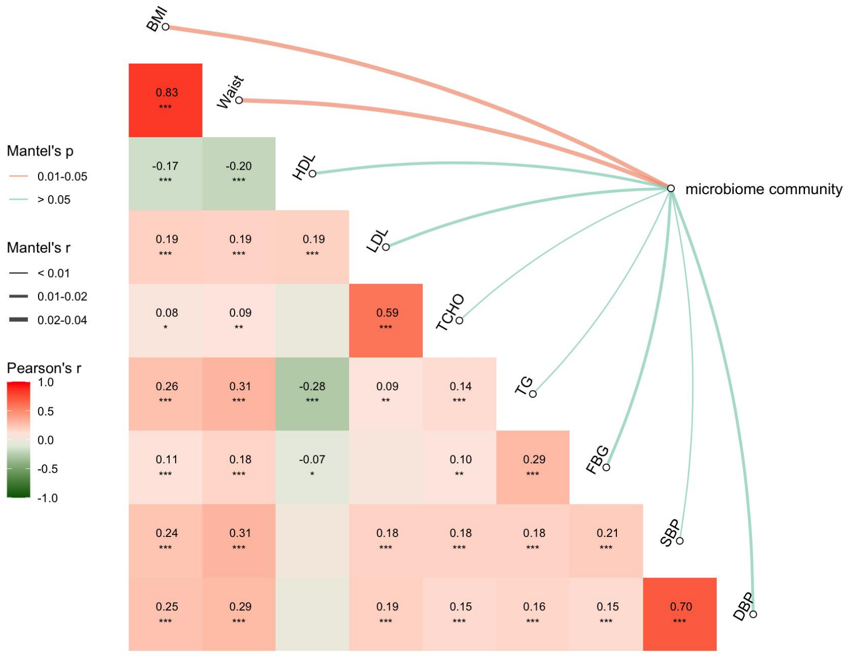

Figure S22: Association analyses for the GGMP dataset. (a) PCoA plots using the Bray–Curtis dissimilarity. (b) Mantel test examining the relationship between gut microbiota and CMD-related risk factors (BMI, WC, HDL, LDL, TCHO, TG, FBG, SBP, and DBP). For subplot (b), $*p < 0.05$, $**p < 0.01$, $***p < 0.001$.

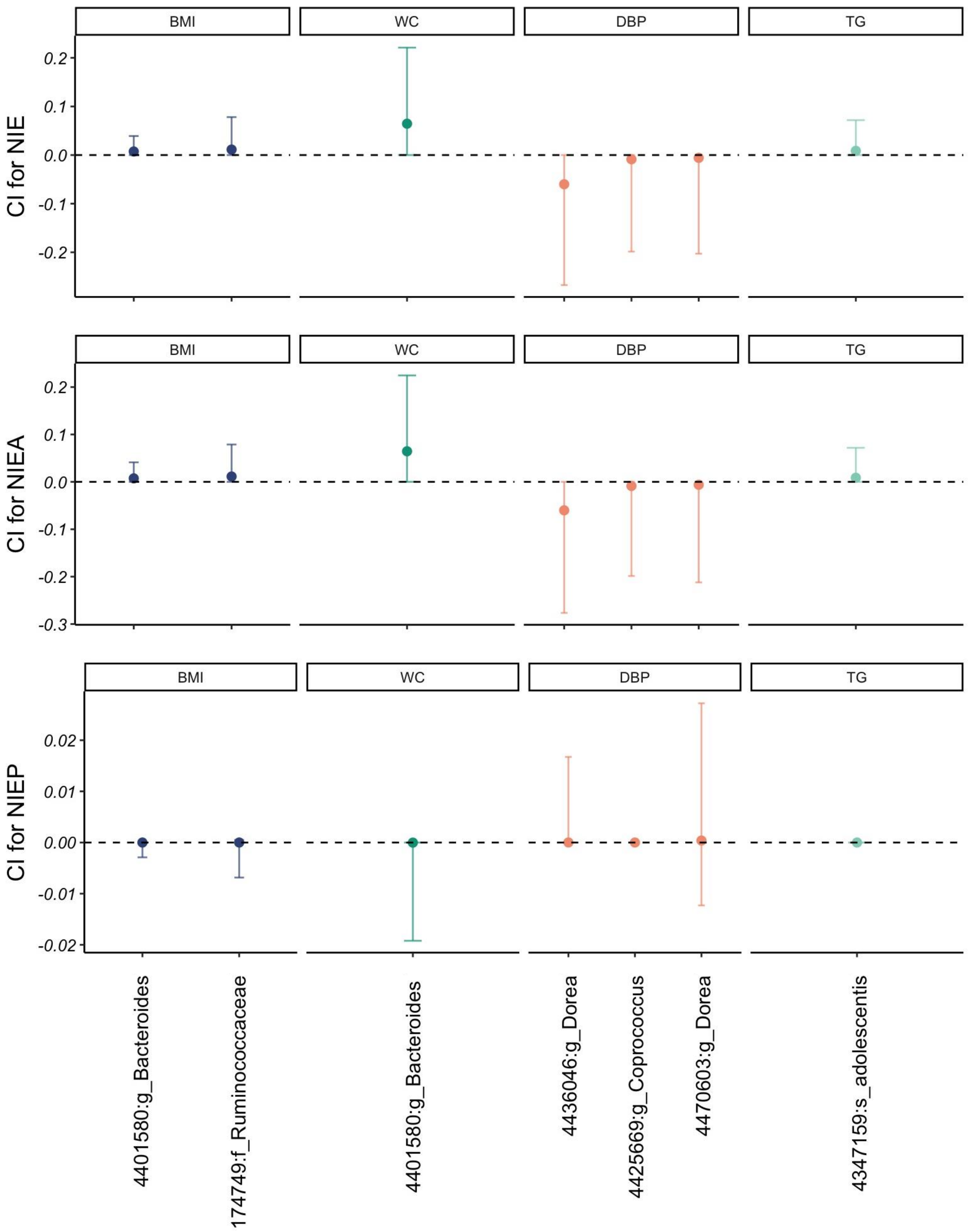

Figure S23: Point and 95% CI estimates of NIE, NIEA, and NIEP for CRAmed-identified OTUs for the GGMP dataset mediating the effect of antibiotics on CMD-related risk factors. 95% CI estimates of NIE, NIEA, and NIEP were calculated based on the permutation strategy with 1000 repetitions.

References

[1] Moran Yassour, Tommi Vatanen, Heli Siljander, Anu-Maaria Hämäläinen, Taina Härkönen, Samppa J Ryhänen, et al. Natural history of the infant gut microbiome and impact of antibiotic treatment on bacterial strain diversity and stability. *Science Translational Medicine*, 8(343):343ra81, 2016.

[2] Yan He, Wei Wu, Hui-Min Zheng, Pan Li, Daniel McDonald, Hua-Fang Sheng, et al. Regional variation limits applications of healthy gut microbiome reference ranges and disease models. *Nature Medicine*, 24(10):1532–1535, 2018.
